# Chromatin accessibility plays a key role in selective targeting of Hox proteins

**DOI:** 10.1186/s13059-019-1721-4

**Published:** 2019-06-03

**Authors:** Damiano Porcelli, Bettina Fischer, Steven Russell, Robert White

**Affiliations:** 1 Department of Physiology, Development and Neuroscience, University of Cambridge, Cambridge, CB2 3DY UK; 2 Department of Genetics, University of Cambridge, Cambridge, CB2 3EH UK; 3 Cambridge Systems Biology Centre, University of Cambridge, Cambridge, CB2 1QR UK

**Keywords:** Hox gene, Hox protein, Chromatin, Chromatin accessibility, Hox cofactors, Transcription factor, Transcription factor selectivity

## Abstract

**Background:**

Hox transcription factors specify segmental diversity along the anterior-posterior body axis in metazoans. While the different Hox family members show clear functional specificity in vivo, they all show similar binding specificity in vitro and a satisfactory understanding of in vivo Hox target selectivity is still lacking.

**Results:**

Using transient transfection in Kc167 cells, we systematically analyze the binding of all eight *Drosophila* Hox proteins. We find that Hox proteins show considerable binding selectivity in vivo even in the absence of canonical Hox cofactors Extradenticle and Homothorax. Hox binding selectivity is strongly associated with chromatin accessibility, being highest in less accessible chromatin. Individual Hox proteins exhibit different propensities to bind less accessible chromatin, and high binding selectivity is associated with high-affinity binding regions, leading to a model where Hox proteins derive binding selectivity through affinity-based competition with nucleosomes. Extradenticle/Homothorax cofactors generally facilitate Hox binding, promoting binding to regions in less accessible chromatin but with little effect on the overall selectivity of Hox targeting. These cofactors collaborate with Hox proteins in opening chromatin, in contrast to the pioneer factor, Glial cells missing, which facilitates Hox binding by independently generating accessible chromatin regions.

**Conclusions:**

These studies indicate that chromatin accessibility plays a key role in Hox selectivity. We propose that relative chromatin accessibility provides a basis for subtle differences in binding specificity and affinity to generate significantly different sets of in vivo genomic targets for different Hox proteins.

**Electronic supplementary material:**

The online version of this article (10.1186/s13059-019-1721-4) contains supplementary material, which is available to authorized users.

## Background

Although in vitro studies of transcription factor-DNA interactions have provided extensive insight into how transcription factors bind DNA [1–3], we have less understanding of the basis of transcription factor specificity in the context of chromatin, the environment in which they operate in vivo. Our lack of understanding of in vivo transcription factor specificity is exemplified by the generally poor correspondence between in vivo binding sites identified by chromatin immunoprecipitation (ChIP) approaches and predicted target sites based on motifs defined by in vitro studies [4]. Further investigation of the interaction between transcription factors and chromatin is needed to increase our understanding of in vivo transcription factor specificity and improve our ability to predict genomic targets.

A particularly clear example of our inadequate understanding of in vivo targeting of transcription factors is provided by the Hox class of homeodomain proteins. This highly conserved family of transcription factors direct the development of different segmental morphologies along the metazoan anterior-posterior axis, with the classic example of the *Drosophila* Hox gene *Ultrabithorax* (*Ubx*) specifying development of the haltere balancer organ in the third thoracic segment which, in the absence of *Ubx*, develops as a wing (reviewed in [5–7]). Each of the eight *Drosophila* Hox genes directs the development of a different segmental morphology in vivo; however, all of the Hox proteins show very similar DNA binding preferences when assayed in vitro (reviewed in [8]). A potential way out of this conundrum is provided by the cofactors Extradenticle (Exd) and Homothorax (Hth) in *Drosophila* and their vertebrate homologs the Pbx and Meis proteins, which interact with Hox proteins to form a tripartite complex [9–12]. In the presence of these cofactors, Hox proteins show a longer consensus binding site and there is evidence of increased differential binding specificity for different Hox proteins [13–15]. In some cases, the formation of the Hox-cofactor complex changes the binding preference of the Hox protein providing “latent specificity” [16]. However, we still do not have a satisfactory understanding of in vivo Hox specificity since first, it is not clear whether the cofactor-enhanced specificity is sufficient to explain the in vivo targeting of Hox proteins and second, in some situations, such as the classic specification of haltere development described above, Hox proteins function in the absence of Exd/Hth cofactors [17].

Previously, we investigated the binding of selected Hox proteins in the context of chromatin through ChIP followed by high-throughput sequencing (ChIP-Seq) in Kc167 cells [18]. We found a strong influence of chromatin state on Hox binding with Ubx and Abdominal-A (Abd-A) binding almost exclusively to DNase1 accessible chromatin, whereas Abdominal-B (Abd-B) exhibited a different specificity and bound to additional genomic sites. This binding, in the absence of Exd/Hth, demonstrated the ability of Hox proteins to exhibit target specificity in the context of chromatin. In addition, the Abd-B-specific binding sites were predominantly in relatively DNase1 inaccessible chromatin. This suggested that histones, rather than simply forming a block to Hox protein binding and restricting the genomic sequence available for binding, might instead play a role in Hox specificity enabling Abd-B to bind to a distinct set of targets through its ability to compete with nucleosomes.

In this report, we present a more comprehensive analysis of the binding of all eight *Drosophila* Hox proteins in the context of chromatin. We demonstrate that they each show distinct chromatin accessibility profiles and that high selectivity of Hox binding is associated with relatively inaccessible chromatin. In addition, we find that a major role of Exd/Hth cofactors is to promote Hox binding to relatively inaccessible chromatin. Overall, our studies indicate a key role for chromatin accessibility in determining the selective in vivo targeting of the different members of the Hox protein family.

## Results

### Hox protein binding in Kc167 cells

We carried out a systematic in vivo analysis of the genome-wide binding of all eight *Drosophila* Hox proteins using our previously established approach [18] designed to maximize comparability between samples. Briefly, we used transient transfection of *Drosophila* Kc167 cells with inducible expression constructs producing Hox-GFP fusion proteins. The cells were fixed 4 h after expression induction and then we used a fluorescence-activated cell sorter calibrated to select cells with the same range of Hox-GFP fusion protein expression. We estimated the expression range to correspond to 38,000–74,000 Hox-GFP molecules per cell, which is comparable to estimates of in vivo homeodomain protein expression of 20,000–50,000 molecules per nucleus [19–22]. Genome-wide binding profiles were generated by chromatin immunoprecipitation, using an antibody against the GFP tag, followed by high-throughput sequencing (ChIP-Seq). For each Hox protein, we collected at least two biological replicates for subsequent analysis.

**Fig. 1:**
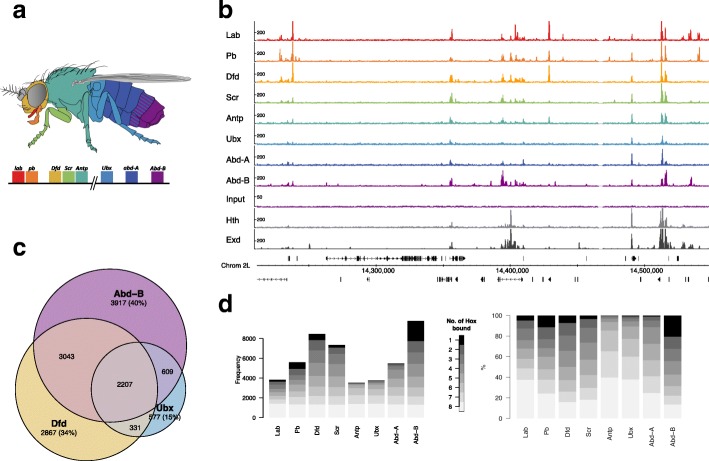
Overview of Hox protein binding in Kc167 cells. **a** Schematic of an adult Drosophila showing domains of deployment of the 8 Hox genes. **b** Representative genomic region showing scaled binding profiles of the 8 Hox proteins and the Hox cofactors Hth and Exd. The Exd profile shows the binding of Exd when expressed in association with Hth. The representative Input profile is from the Abd-B transfection (arbitrary scaling). **c** Venn diagram showing overlap analysis of binding regions (*q* value 1e−2) for selected Hox proteins Dfd, Ubx, and Abd-B. Number in brackets gives the number of non-overlapping regions as a percentage of the total number of regions for each protein. **d** Plots of Hox binding selectivity. For each Hox protein, binding regions (*q* value 1e−2) are classified according to the number of Hox proteins bound (see scale). Plotted on the left as frequencies and on the right normalized as percentages of total peak number for each Hox protein

The binding profiles (Fig. 1) show that all eight Hox proteins have distinct but overlapping sets of genomic binding targets. There is a large variation in the numbers of binding regions identified for the different Hox proteins (846 for Antp to 5685 for Abd-B at q1e^−10^) and also in the proportion of binding regions unique to an individual Hox protein (Fig. 1d). Apart from the centrally expressed Hox proteins, Antp, Ubx, and Abd-A, the Hox proteins each show significant numbers of unique sites, demonstrating that the Hox family does not simply bind to a nested set of targets.

### Motif enrichment

**Fig. 2:**
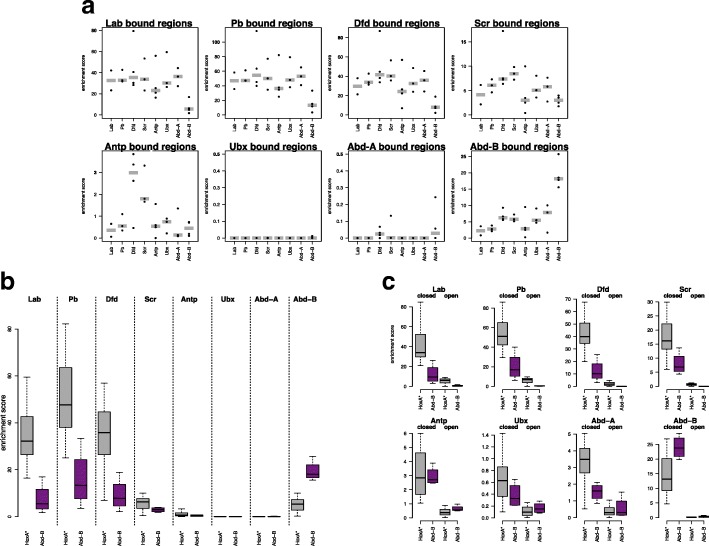
Analysis of Hox binding motifs. **a** Motif enrichment analysis on the top 500 binding regions (200 bp regions using binding summit position extended ± 100bp) for each Hox protein. Plot titles indicate binding region set used, and motifs are indicated on the *x*-axis. Enrichment analysis was performed using PWMEnrich for the Hox motifs in the MotifDb database (see the “Materials and Methods” section for details of the motif sets). Enrichment scores [log_10_(1/*p* value)] for individual motifs are indicated (dots) together with the median for each motif set (grey bar). Note the differences in *y*-axis scales. **b** Motif enrichment analysis on the same binding region sets as in **a** using the merged HoxA* (combining scores for the Lab, Pb, Dfd, Scr, Antp, Ubx, Abd-A motifs; grey) and Abd-B (purple) motifs. Boxplot with horizontal line indicating the median, box indicating upper and lower quartiles, and whiskers indicating the highest and lowest values excluding outliers. **c** Motif enrichment analysis for Hox group peaks separated according to chromatin accessibility, using 500 randomly selected “open” (Kc167 ATAC-Seq *q* < 1e−2) or “closed” regions

To investigate the basis for the distinct Hox binding profiles, we compared the enrichment of in vitro-defined Hox binding motifs for the individual Hox proteins in each set of binding regions (Fig. 2a). This analysis revealed two insights: first, there is wide variation in the general level of Hox motif enrichment, with an anterior group of Hox proteins, Lab, Pb, Dfd, and Scr, showing high enrichments, a central group of Hox proteins, Antp, Ubx, and Abd-A, showing little motif enrichment and the most posterior Hox protein, Abd-B, showing substantial enrichment. Second, with the exception of the Abd-B motif, there is little clear discrimination between the different motifs, i.e., for each binding site set the motifs for Lab to Abd-A exhibit similar levels of enrichment, whereas the Abd-B motif is discriminating, with low enrichment in the anterior Hox binding sets but high enrichment in the Abd-B binding set. The difference between the Lab to Abd-A motifs and the Abd-B motif fits with a clear shift in base preference in the core motif, from TAAT to TTAT [3, 8]. Grouping the motifs on this basis, with Lab to Abd-A motifs grouped as HoxA* (Fig. 2b), provides a simpler view of the enrichment data emphasizing that the three most anterior Hox proteins exhibit much stronger enrichment than the others and demonstrating a clear switch in preference between Lab, the most anterior Hox, and the most posterior, Abd-B. In addition, we observed a trend in the shift from HoxA* to Abd-B motif preference across the whole Hox set.

**Fig. 3:**
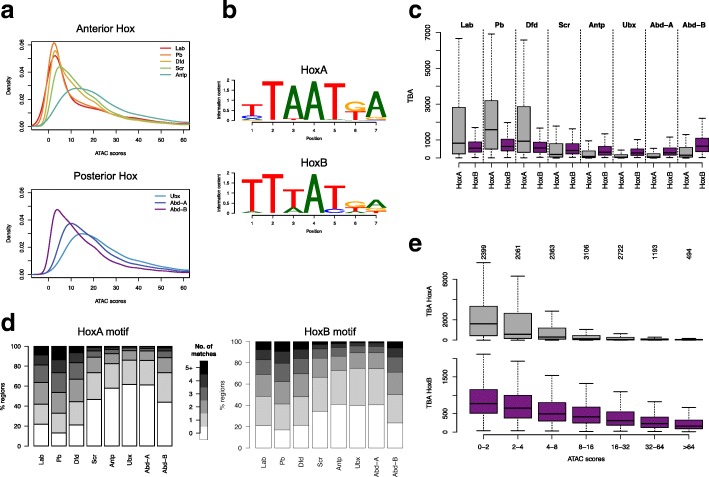
Roles of chromatin accessibility and affinity in Hox binding. **a** Density plots of mean ATAC-seq scores for Hox group peak regions (200 bp regions). Left plot anterior Hox proteins, right plot posterior Hox proteins. **b** HoxA (consensus motif combining Lab, Pb, Dfd, Scr, Antp, Ubx, Abd-A motifs) and HoxB motifs. **c** Boxplot of the total binding affinity (TBA) for HoxA (grey) and HoxB (purple) motifs for the top 500 binding regions for each Hox protein. **d** Plots of Hox group peak regions for each Hox protein classified according to the number of motif matches (HoxA on left, HoxB on right) they contain (see scale). For each Hox protein, matches were counted in the top 500 (by ChIP score) Hox group peak regions using the matchPWM function in Biostrings R package with min.score = 90%. **e** Boxplot showing relationship of TBA and chromatin accessibility, with HoxA (upper) and HoxB (lower) TBA for regions bound by any Hox binned by ATAC score

Since we have previously shown that Hox motif enrichment and chromatin accessibility are linked [18], we analyzed the motif enrichments separately for “open” and less accessible “closed” chromatin to investigate the wide variation in general Hox motif enrichment observed. For this, we classified Hox binding regions based on ATAC-Seq scores from untransfected Kc167 cells. We found a dramatic difference in enrichment scores between open and closed chromatin (Fig. 2c). Hox binding sites in open chromatin show little enrichment for Hox motifs, whereas high levels of enrichment are found in closed chromatin, particularly for the anterior Hox proteins, Lab, Pb, Dfd, and Scr, and for the most posterior Hox, Abd-B. This suggests that the variation in general Hox motif enrichment for the different Hox binding site sets may be linked to the propensity for each Hox protein to bind less accessible chromatin. We therefore examined the chromatin accessibility distribution of the binding sites of the different Hox proteins (Fig. 3a) and found a strong concordance with the motif enrichment levels. The Hox proteins with the higher motif enrichments, Lab, Pb, Dfd, Scr, and Abd-B, bind predominantly to “closed” chromatin, whereas those with low motif enrichment, Antp, Ubx, and Abd-A, bind predominantly to open chromatin. In addition, the chromatin accessibility distributions show interesting progressions. Anteriorly from Antp and posteriorly from Ubx the Hox proteins present a sequence of increasing binding to less accessible chromatin. These progressions provide an intriguing link between the domains of action of Hox proteins along the body axis and their binding to chromatin.

Another way to characterize binding sites is through their total binding affinity (TBA; [23, 24]), scanning each binding region to produce a cumulative score based on both the number and quality of motif matches. For this analysis, we combined the PWMs (using the motifs from the JASPAR database) for Lab to Abd-A to give the composite HoxA PWM and we renamed the PWM for Abd-B as HoxB (Fig. 3b). Similar to the situation with motif enrichment, a clear correspondence between TBA and chromatin accessibility distribution is seen. The binding sites for Hox proteins that bind to less accessible chromatin (Lab, Pb, Dfd, and Abd-B) show high TBA for their preferred motifs, whereas the binding sites for Hox proteins that bind predominantly open chromatin show low TBA (Fig. 3c). In addition, the TBA shows a clear switch in Hox motif preference from HoxA to HoxB between anterior Hox proteins and Abd-B, as well as a trend in preference switching across the whole Hox set (Fig. 3c). The high TBA scores are based on both the quality and number of motif matches in each 200 bp binding region (Fig. 3d). The general relationship between TBA and chromatin accessibility for both HoxA and HoxB motifs shows a clear inverse relationship to accessibility (Fig. 3e).

Overall, this analysis shows the relevance of both specific binding affinity, based on the quality and quantity of preferred motifs in binding regions, and chromatin accessibility for Hox protein target site selection. Binding to closed chromatin is associated with high TBA that may enable Hox proteins to effectively compete with nucleosomes. In addition, Hox binding occurs across a range of chromatin accessibility and here competition with chromatin may provide the potential for subtle differences in motif preference to generate different target sets for particular Hox proteins.

### Hox selectivity

**Fig. 4:**
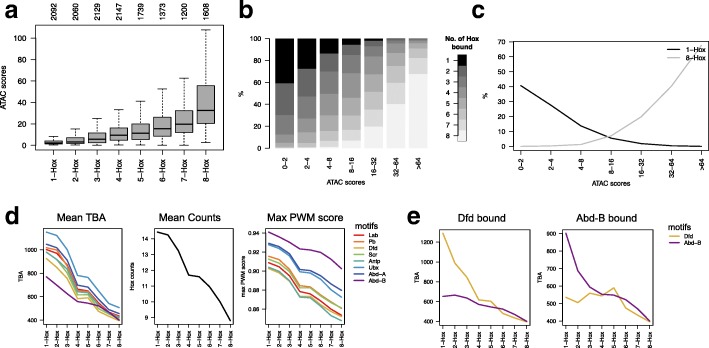
Roles of chromatin accessibility and affinity in Hox selectivity. **a** Boxplot showing relationship of Hox selectivity to chromatin accessibility (ATAC score). Hox selectivity is represented by binning Hox group peak regions according to the number of Hox proteins bound; 1-Hox: only 1 Hox protein bound; 8-Hox: all 8 Hox proteins are bound. Number of regions in each Hox selectivity bin is shown above the plot. **b** Plot showing relationship of Hox selectivity classes to chromatin accessibility. The Hox group peaks are then separated into ATAC score bins and the frequency of Hox selectivity classes (see scale) is plotted. **c** Plot showing opposing distributions of sites binding all Hox proteins (8-Hox) and uniquely bound sites (1-Hox) with respect to chromatin accessibility (ATAC score bins). **d** Plots showing relationship between Hox selectivity and (from left) TBA (for each 7-mer Hox PWM from the JASPAR database), mean number of occurrences of Hox motifs (using matchPWM function with min.score = 80%) and highest PWM match score within each region for binding regions as in **a**. **e** Relationship between Hox selectivity and TBA for sets of binding regions for particular Hox proteins. Left for the regions bound by Dfd and right for the regions bound by Abd-B, plotting the TBAs for the Dfd (orange) and Abd-B (purple) motifs

We next examined the relationship between chromatin accessibility and the selectivity of Hox binding, as measured by the number of different Hox proteins binding to any particular region. We found a clear relationship, supporting a key role for chromatin accessibility in Hox selectivity. As shown in Fig. 4a–c, increasing selectivity is associated with decreasing chromatin accessibility. Sites showing highest Hox selectivity, binding only one member of the Hox protein family, are concentrated in less accessible chromatin whereas sites in open chromatin tend to be poorly discriminating, binding several different Hox proteins. The relationship is gradual with the progressive increase in Hox selectivity associated with decreasing chromatin accessibility across the range of ATAC-Seq scores.

Hox selectivity is positively correlated with Hox TBA as seen in the general relationship between Hox selectivity and TBA for the individual Hox binding motifs (Fig. 4d). The increasing binding region affinity with increasing Hox selectivity reflects both increasing affinity of individual binding sites (measured by the quality of match to a position weight matrix (PWM)) and an increasing number of Hox binding sites within the binding region. In addition, high selectivity for a particular Hox protein is associated with differential TBA for preferred binding motifs. This is illustrated by comparing the target sets for Dfd and Abd-B (Fig. 4e). For the low selectivity sites, the TBA plots are similar; however, for the higher selectivity sites, on the left of the plots, the TBA values show specific inflection; for Dfd sites, there is a specific rise in TBA for the Dfd motif, whereas TBA for the Abd-B motif remains relatively flat. For the Abd-B target set, the reverse occurs. Additional file 1: Figure S1a shows further analysis of the relationship between Hox selectivity and binding region affinity, with subsets selected according to chromatin accessibility.

Overall, the association of high Hox selectivity with relatively inaccessible chromatin and high-affinity binding regions indicates an interplay between affinity and chromatin accessibility in enabling Hox proteins to bind to different target sets. In a model of competition between nucleosomes and Hox proteins, binding to less accessible chromatin requires a higher affinity interaction between the Hox protein and the binding region. Accordingly, we find Hox binding regions in open chromatin show little discrimination, binding several or all Hox proteins. However, in less accessible chromatin, where competition with nucleosomes provides a basis for selective binding based on affinity, binding regions show more discrimination.

### Roles of the canonical Hox cofactors, Exd and Hth

In many situations, Hox proteins bind in association with the canonical Hox cofactors, Exd and Hth [8, 10]. To examine the roles of Exd/Hth in Hox binding, we systematically expressed Hth in bicistronic constructs with each GFP-tagged Hox protein and generated ChIP-Seq binding profiles for the Hox proteins as described above. Kc167 cells lack Hth but do express Exd, which is cytoplasmic in the absence of Hth. Expression of Hth recruits Exd into the nucleus and provides Exd/Hth cofactor function [18].

**Fig. 5:**
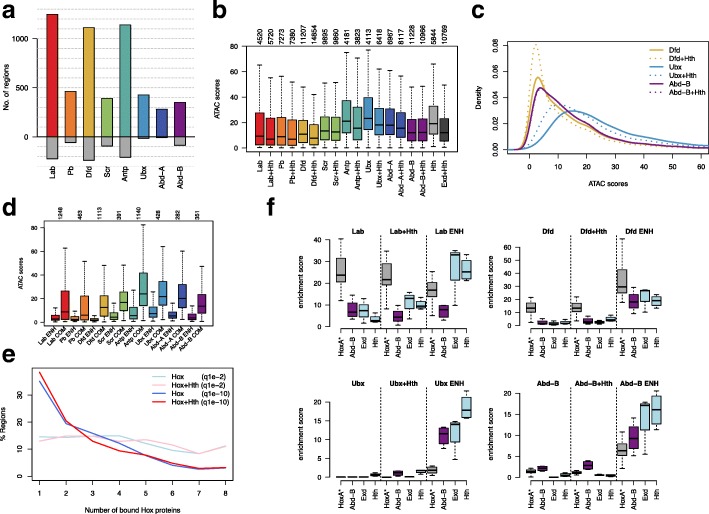
Effect of Exd/Hth on Hox binding. **a** Plot showing the number of cofactor-enhanced binding regions based on differential binding (fdr ≤ 0.01, logFC ≥ 1 and both replicates bound at macs q1e−2). Regions more bound in the presence of Exd/Hth (Hth+Hox) are shown in colour as positive numbers, regions more bound in Hox alone compared to Hth+Hox are shown in grey underneath. **b** Boxplot of ATAC scores in Hox group peaks for Hox proteins in the absence (Hox) or presence (Hox+Hth) of Exd/Hth. The Hth regions are bound by Hth-GFP and the Exd+Hth regions are bound by Exd-GFP in the presence of Hth. Numbers of bound regions are indicated above the plot. **c** Density plots of mean ATAC-seq scores for Hox group peaks bound by Dfd, Ubx, and Abd-B with and without Exd/Hth showing the effect of the cofactors on the chromatin accessibility profile. Solid lines: Hox alone, dotted lines: Hox in presence of Exd/Hth. **d** Boxplot comparing chromatin accessibility of Exd/Hth enhanced regions (Hox ENH) versus common regions (bound similarly in the presence or absence of Exd/Hth; Hox COM). Numbers for Hox ENH regions are given above the plot and the same number of randomly selected common regions was used for Hox COM. **e** Plot showing lack of effect of Exd/Hth on the Hox selectivity profile plotting percentage of regions in each of the Hox selectivity classes for Hox alone (Hox) and in the presence of Exd/Hth (Hox+Hth). This lack of effect is a robust observation across both low (q1e−2) and high (q1e−10) stringency binding regions. **f** Motif analysis comparing motif enrichment for binding regions for Hox alone (Hox), Hox in the presence of Exd/Hth (Hox+Hth), and Exd/Hth cofactor enhanced binding regions (Hox ENH) using 500 randomly selected regions from each class for selected Hox proteins Lab, Dfd, Ubx and Abd-B. Motifs are HoxA* (grey; see Fig. 2), Abd-B (purple, see Fig. 2), and the Exd and Hth motifs (light blue)

The addition of Exd/Hth generally promotes Hox binding, and although the simple thresholded peak counts are not always increased (Additional file 1: Figure S1c), differential binding analysis revealed a significant set of cofactor-enhanced regions for all the Hox proteins (Fig. 5a). Since we previously showed that Exd/Hth increases the ability of Ubx to bind to closed chromatin [18], we examined the effect of Exd/Hth on the chromatin accessibility distribution. For all Hox proteins, apart from Abd-B (and with only a minimal shift in the case of Scr), the chromatin accessibility profile is shifted towards lower ATAC-Seq scores indicating that the provision of Exd/Hth enables Hox proteins to bind to less accessible chromatin (Fig. 5b, c; Additional file 1: Figure S1d and S2). Comparing sites where the presence of cofactors results in significantly enhanced binding (cofactor-enhanced sites) with sites which bind Hox but whose Hox binding does not change significantly in the presence of Exd/Hth (common sites) revealed a clear difference in chromatin accessibility. The common sites are predominantly in open chromatin whereas the cofactor-enhanced sites are generally in closed chromatin, and for all Hox proteins, there is a clear decrease in median ATAC-Seq score for the cofactor-enhanced sites compared with the common sites (Fig. 5d).

**Fig. 6:**
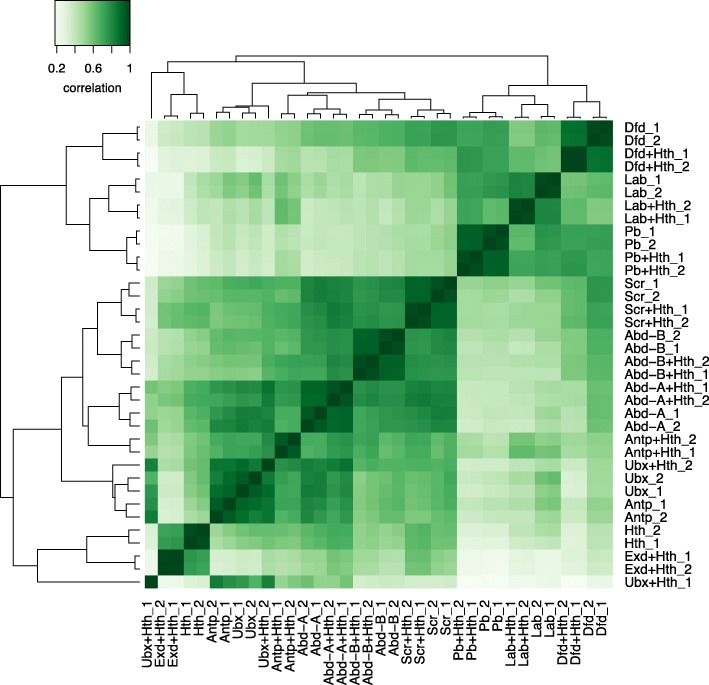
Hox specificity is expressed independently of Exd/Hth. Correlation heatmap of ChIP-Seq reads showing general clustering together of individual Hox and Hox+Hth samples. Reads were counted overlapping 20 bp windows of the union of macs q1e−2 bound regions across all Hox and Hox+Hth samples (for details see the “Materials and Methods” section)

Although there is considerable in vitro evidence that Exd/Hth can increase the specificity of Hox binding [13–16], their role in vivo is less clear. As illustrated above, Hox proteins can bind to distinct sets of genomic targets in vivo in the absence of Exd/Hth. Cluster analysis based on ChIP-Seq reads provides a global view, showing that individual Hox binding profiles cluster separately from one another and, strikingly, Hox plus Exd/Hth profiles cluster together with their respective Hox; e.g., Dfd and Dfd+Hth cluster together and separately from Lab and Lab+Hth (Fig. 6). This demonstrates that Hox proteins display clear individual specificities in vivo independent of Exd/Hth. We also note from this analysis that the anterior Hox proteins Lab, Pb, and Dfd show a close association, clustering together and distinct from the remaining Hox proteins which fits with their grouping on the basis of high motif enrichment in their binding regions (Fig. 2b).

We assessed the global effect of Exd/Hth on Hox target selectivity by examining the cofactor effect on the distribution of regions according to the number of different Hox proteins they bind. We found that this Hox discrimination profile is little changed by the presence of Exd/Hth (Fig. 5e). We also examined the effect of Exd/Hth on the motif enrichment profiles (Fig. 5f). In general, the enrichment profile for the Hox motifs is little affected by the addition of Exd/Hox although there are clear increases in Exd and Hth motif enrichment. In the Antp, Ubx, and Abd-A target sets, the provision of Exd/Hth enhances the relative enrichment of the Abd-B motif above the others and this may represent the latent specificity effect of Hox/Exd dimer binding [16] (see also Additional file 1: Figure S3).

**Fig. 7:**
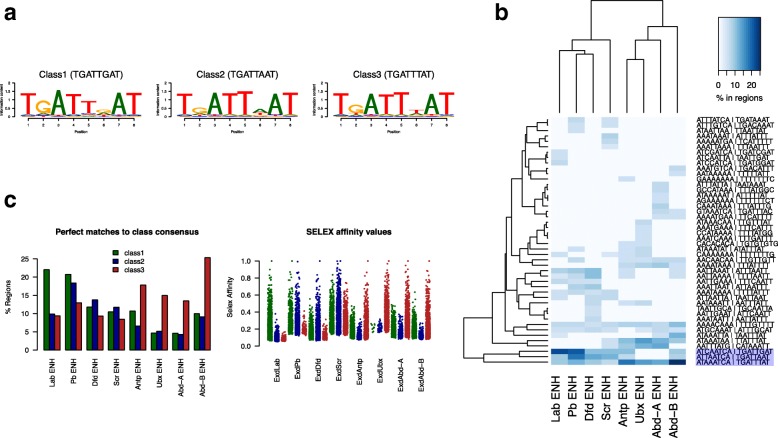
In vivo motif preferences for Hox binding in the presence of Exd/Hth. **a** Constrained pattern matching on in vivo binding regions defines 3 classes of consensus sequences. Matches to the pattern TGATTDAT (where D = A or G or T), based on the in vitro SELEX Exd-Hox sites [16], in defined sets of binding regions were used to create the three matrices. The binding regions used were class 1 unique Exd/Hth enhanced Lab bound, class 2 unique Exd/Hth enhanced Pb or Dfd or Scr bound, and class 3 unique Exd/Hth enhanced Antp or Ubx or Abd-A or Abd-B bound. The pattern matching allowed one mismatch. **b** The Class 1, 2 and 3 consensus sequences (highlighted) are the most enriched 8-mers in unbiased *k*-mer enrichment analysis on Exd/Hth-enhanced Hox binding regions (Hox ENH). Enrichment of the top 15 *k*-mers in each binding region set is plotted as a heatmap. **c** Correspondence of in vivo and in vitro binding specificities. The left plot shows the percentage of regions from the Exd/Hth-enhanced Hox binding regions with perfect matches to the class 1, 2, and 3 consensus sequences. The right plot shows the affinity scores for SELEX 16-mers which contain the three Class consensus sequences for the different SELEX Hox+Exd experiments in Slattery et al. [16]; class 1: green, class 2: blue; class 3: red

We performed de novo motif finding analysis on sites where the cofactors significantly increase binding (Additional file 1: Figure S4). Combining the most similar motifs, led to the identification of three classes of cofactor-Hox PWMs (Fig. 7a). A *k*-mer analysis shows that these consensus sequences are the most enriched *k*-mers in the cofactor-enhanced binding regions (Fig. 7b). Preference between these three PWMs provides a clear view of the graded motif preferences across the whole set of eight Hox proteins. Strikingly, these in vivo derived preferences correspond extremely well with the preferences defined by in vitro SELEX analysis of Hox binding in association with Exd [16] (Fig. 7c).

Overall, we find that Exd/Hth has significant effects on in vivo Hox binding; for example, almost doubling the number of Dfd-bound regions (increasing from 4782 to 8958 peaks at q1e^−10^). The cofactors increase the length of the enriched binding motifs and facilitate Hox protein binding to less accessible chromatin.

### Hox binding and chromatin accessibility

**Fig. 8:**
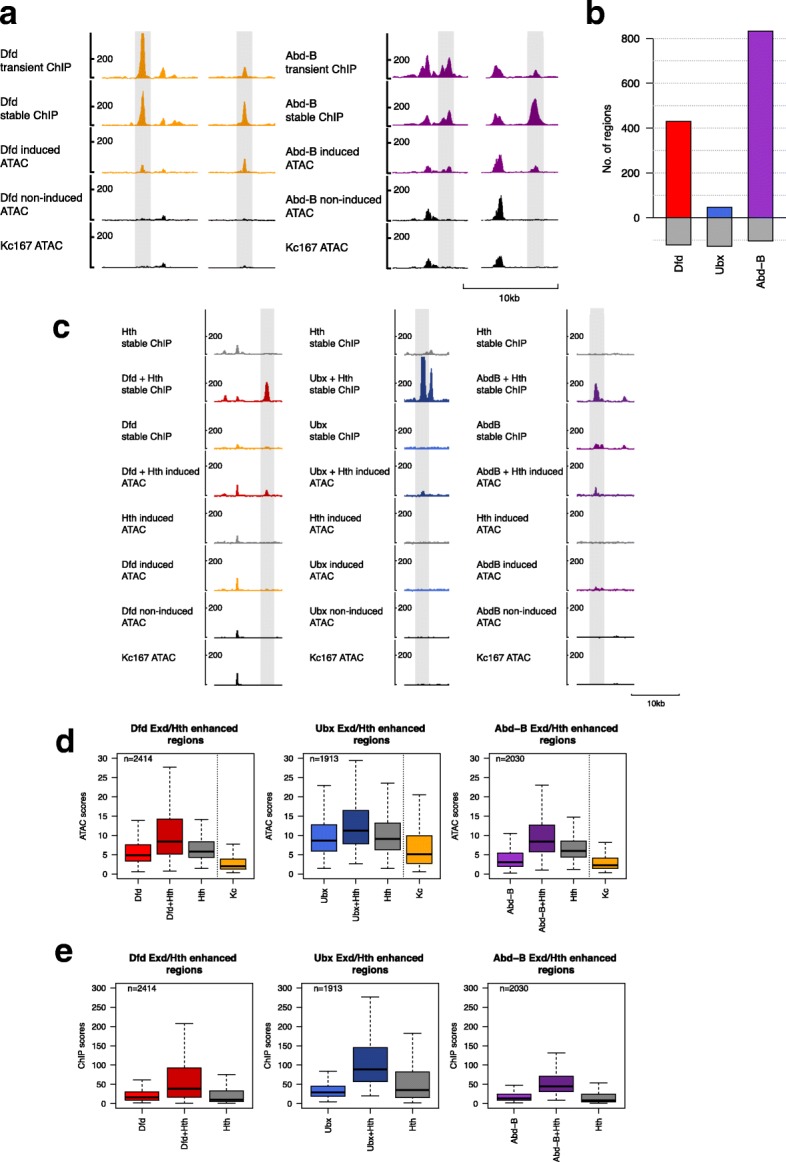
Hox proteins collaborate with Exd/Hth promoting chromatin accessibility. **a** Representative ChIP-Seq and ATAC-Seq profiles showing increased chromatin accessibility on Dfd and Abd-B binding. **b** Number of regions with significantly increased chromatin accessibility (edgeR fdr ≤ 0.01 and logFC≥ 1.5 for ATAC-Seq reads) on induced versus non-induced samples for Dfd, Ubx, and Abd-B are shown as colored bars. Number of regions with significantly reduced ATAC-Seq reads (edgeR fdr ≤ 0.01 and logFC ≤ − 1.5 for ATAC-Seq reads) are shown in grey as negative values. **c** Representative ChIP-Seq and ATAC-Seq profiles showing collaboration between Hox and Exd/Hth promoting chromatin accessibility. **d** Boxplot of ATAC scores in Exd/Hth-enhanced Hox binding regions for stable lines expressing Hox alone, Hox in the presence of Hth (Hox+Hth), Hth alone and, as a reference, the basal Kc167-cell (Kc) ATAC scores. All three Hox+Hth show increased ATAC scores compared to either Hox alone or Hth alone; *p* values < 0.01, Dunn’s Kruskal-Wallis multiple comparison. Although the Kc167 ATAC scores cannot be directly compared to the stable cell line ATAC score data, the low median scores indicates that these regions are relatively inaccessible in the basal Kc167 state. **e**: Boxplot of ChIP-seq scores in the same regions as in **d** showing Hox ChIP, Hox ChIP in the presence of Exd/Hth (Hox+Hth) and Hth ChIP

To understand better the link between chromatin accessibility and Hox binding, we investigated the effects of Hox binding on accessibility and also the effects of other transcription factors that either promote Hox binding or are known to be able to open chromatin (so-called pioneer factors). To study effects on chromatin accessibility using ATAC-Seq, we generated stable cell lines expressing representative Hox proteins, Dfd, Ubx, and Abd-B, since the transient transfections were not suitable for ATAC-Seq due to the dominance of plasmid sequences in the ATAC-Seq libraries. We compared the ATAC-Seq profiles of induced versus non-induced cell lines and found clear evidence that Hox proteins vary in their propensity to open chromatin. We see chromatin opening by Dfd and Abd-B (Fig. 8a) but not by Ubx. Differential peak analysis confirms that Dfd and Abd-B demonstrate robust chromatin opening, with the generation of 430 and 832 significantly enhanced ATAC-Seq peaks respectively (at log fold change > 1.5) whereas Ubx shows very little evidence of opening (Fig. 8b and Additional file 1: Table S5).

To investigate the role of Exd/Hth, we generated stable cell lines expressing Dfd, Ubx, or Abd-B proteins together with Hth and found that the cofactors promote chromatin opening (Fig. 8c). Examining the regions with enhanced Hox binding in the presence of Exd/Hth showed that addition of the cofactors increases the median ATAC-Seq score in partnership with each of the three Hox proteins (Fig. 8d). In contrast, when Hth is expressed in the absence of a Hox protein, these regions show low chromatin accessibility and little evidence of Hth binding (Fig. 8e), indicating that the Hox proteins and Exd/Hth work in collaboration to open chromatin. Differential peak analysis on the 21,002 Hox group peaks (stable) regions, comparing induced versus non-induced for the Hth stable cell line, supports the lack of significant chromatin opening when Hth is expressed in the absence of Hox proteins (Additional file 1: Table S5).

**Fig. 9:**
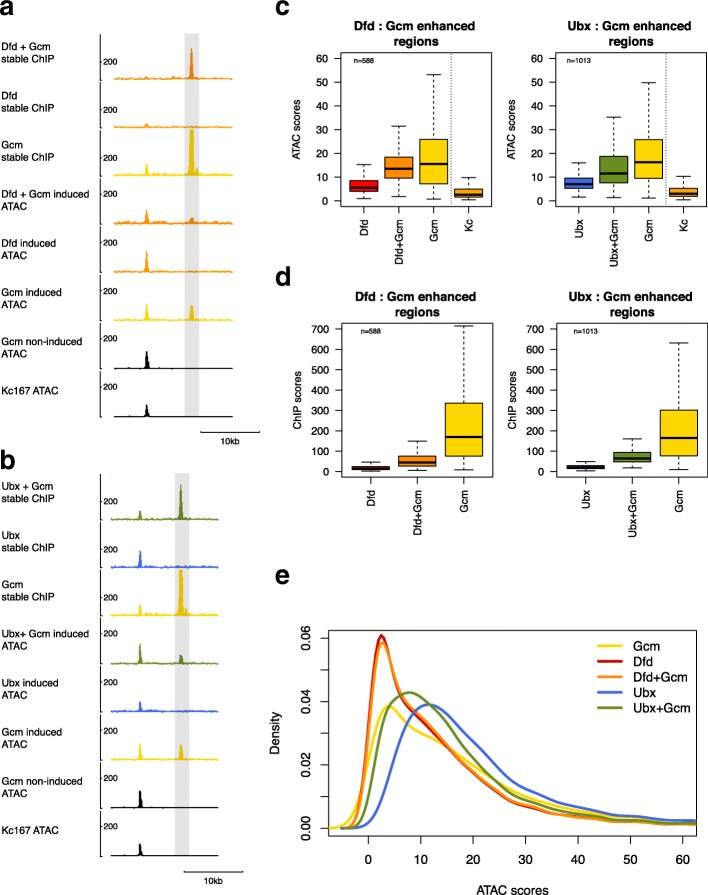
Gcm acts as a pioneer factor promoting Hox binding. **a** Representative ChIP-Seq and ATAC-Seq profiles showing chromatin opening by Gcm and promotion of Dfd binding. **b** Representative ChIP-Seq and ATAC-Seq profiles showing chromatin opening by Gcm and promotion of Ubx binding. **c** Boxplot of ATAC scores in Gcm-enhanced Hox binding regions for Hox, Hox in the presence of Gcm (Hox+Gcm), Gcm and, as a reference, the basal Kc167-cell (Kc) ATAC scores. Both Hox+Gcm show increased ATAC scores compared to Hox alone; *p* values < 0.01, Dunn’s Kruskal-Wallis multiple comparison. The high median ATAC scores for Gcm show that these regions are generally open in the presence of Gcm alone. Although the Kc167 ATAC scores cannot be directly compared to the stable cell line ATAC score data, the low median scores indicates that these regions are relatively inaccessible in the basal Kc167 state. **d** Boxplot of ChIP-seq scores in the same regions as in **c** showing Hox ChIP, Hox ChIP in the presence of Gcm (Hox+Gcm), and Gcm ChIP*.* **e** Density plots of mean ATAC-seq scores for 200 bp Hox group peak regions (stable)

We directly examined the effect of opening chromatin on Hox binding by co-expressing Hox proteins with the hemocyte lineage-determining factor Glial cells missing (Gcm), which is believed to act as a pioneer factor [25]. Kc167 cells show characteristics of hemocytes, which in the in vivo lineage are induced to differentiate into plasmatocytes by Gcm [26, 27]. We first established, by ChIP-Seq and ATAC-Seq in stable Kc167 cell lines expressing Gcm-GFP, that Gcm binding is associated with chromatin opening (Fig. 9a–d). We then expressed Gcm in conjunction with Hox proteins in stable cell lines, choosing Dfd as a representative Hox protein that shows substantial ability to bind to sites in closed chromatin and Ubx, representing Hox proteins whose binding is largely restricted to open chromatin. We found that the presence of Gcm leads to novel binding sites for both Dfd and Ubx (Fig. 9a, b), providing a direct experimental demonstration of the role of chromatin accessibility in Hox target selection. For Dfd, the provision of Gcm generates 1168 novel sites (at q1e^−2^, 13% of the total Dfd binding sites in the presence of Gcm), whereas for Ubx, Gcm has a larger effect, generating 4291 novel sites (49% of the total Ubx binding sites in the presence of Gcm). The smaller effect of Gcm on Dfd binding may reflect the ability of Dfd to bind to less accessible regions on its own and we find that the presence of Gcm has little effect on the accessibility profile of Dfd-bound regions in comparison to the large effect for Ubx (Fig. 9e). Comparison of the sites bound exclusively by either Dfd or Ubx in the presence of Gcm reveals higher TBA, higher motif counts, and higher motif enrichment for the Dfd sites supporting the importance of multiple motifs for Dfd binding (Additional file 1: Figure S8).

Comparing the effects of Exd/Hth versus Gcm on Hox binding reveals two rather different routes to enhance Hox binding. In contrast to the Exd/Hth situation, the sites with significantly increased Hox binding in the presence of Gcm are associated with robust Gcm binding and chromatin opening by Gcm when expressed in the absence of Hox (Fig. 9c and Additional file 1: Figure S5). Thus, while Exd/Hth and Hox work together to enhance chromatin accessibility, Gcm is able to open chromatin independently of Hox and thereby facilitate Hox binding.

### Comparison of chromatin accessibility and binding site affinity in Hox target selection

**Fig. 10:**
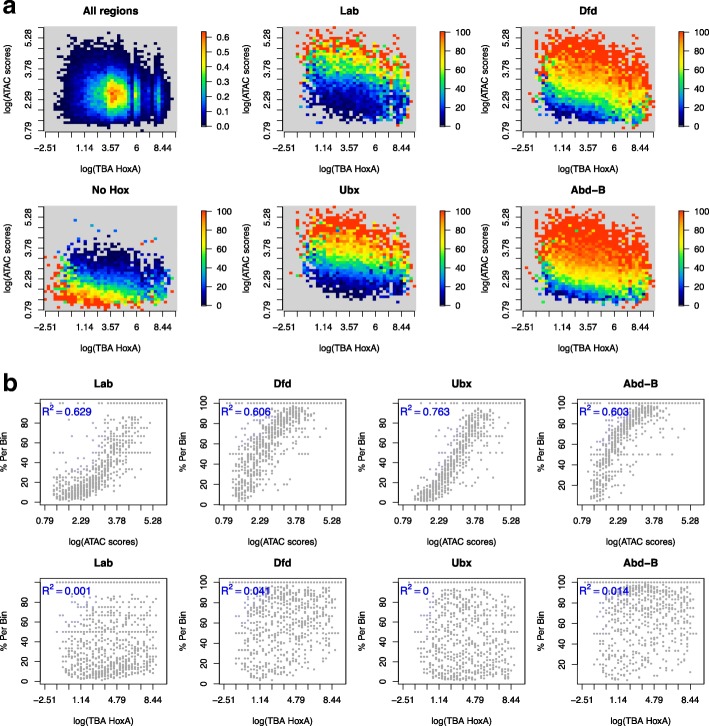
Hox occupancy is more strongly associated with binding region chromatin accessibility than with binding affinity**. a** Scatter plots of chromatin accessibility (log[ATAC scores]) versus binding affinity (log[TBA HoxA]) for chromatin regions classified as “open.” Open chromatin regions were divided into 200bp tiles and the mean ATAC score and TBA for HoxA PWM calculated per tile. The log of these scores was then linearly binned into 40 bins on each axis. For the “All regions” plot, the heatmap shows the density distribution. For the other plots, the heatmap shows the percentage of tiles bound by the specified Hox protein per bin or for “No Hox” the percentage of tiles not bound by any Hox protein. Note that bins with zero percent are given background colour in the heatmap scale. The plots are shown for selected Hox proteins and for TBA for the HoxA PWM (for a fuller set of plots including HoxB TBA, see Additional file 1: Figure S6). **b** Scatter plots show the strong correlation of occupancy (% per bin) with chromatin accessibility (log[ATAC scores]; upper row) and the poor correlation with binding affinity ((log[TBA HoxA]; lower row). Data as in **a**. Further plots in Additional file 1: Figure S6

To gain an overview of the relationships between accessibility, binding site affinity, and Hox occupancy, we plotted the Hox binding data for “open chromatin” as a heatmap of occupancy (percentage of Hox-occupied 200bp open chromatin regions per bin) on a scatter plot of accessibility (logATAC score) versus affinity (logTBA) (Fig. 10a, Additional file 1: Figure S6). Even within these “open” chromatin regions, we see a strong influence of accessibility: regions with low ATAC scores show low occupancy while the most open regions exhibit very high occupancy. In contrast, the correlation between occupancy and TBA is much less strong (Fig. 10b). The relevance of relative accessibility for Hox binding is emphasized by the “No Hox” plot where the heatmap illustrates the percentage of regions not bound by any Hox protein (Fig. 10a). The regions with the least accessibility are associated with no Hox binding, with a graded decrease in unbound regions as the ATAC scores rise. The strong correlation between Hox binding and chromatin accessibility and the observation that the most open regions show close to 100% occupancy suggests that while there is a requirement for openness, there is not a requirement for specific binding partners at the bound open regions.

## Discussion

Ever since the initial analyses of DNA binding by Hox proteins in the 1990s [14, 15, 28, 29], our understanding of the basis for Hox specificity has faced the conundrum that while Hox proteins exhibit clear functional specificity in vivo, the different members of the Hox family show very similar DNA binding specificity in vitro. One of the unknowns for our understanding of in vivo Hox specificity has been the effect of chromatin on Hox binding. Our investigations into Hox protein binding in the context of chromatin in Kc167 cells reveal a strong interplay between target selection by Hox proteins and chromatin accessibility. We find that Hox selectivity shows a graded relationship to chromatin accessibility, with sites in relatively closed chromatin showing highly selective Hox binding while sites in open chromatin tend to be unselective and exhibit binding by most or all Hox proteins (Fig. 4). The binding regions for different Hox proteins show different chromatin accessibility profiles. Some Hox proteins, such as Dfd and Abd-B, bind relatively closed chromatin, whilst others, such as Antp and Ubx, bind almost exclusively to open chromatin (Fig. 3). Binding regions in relatively inaccessible chromatin are generally high affinity, with multiple good matches to consensus binding sites, while binding sites in open chromatin tend to have low affinity (Fig. 3). Our data fit with a model of in vivo Hox specificity based on the different ability of specific Hox proteins to compete with chromatin and access their DNA binding sites.

The propensity to compete with chromatin could depend on a variety of factors. The link between high TBA and high selectivity indicates that target selectivity could depend on differences in the affinity of interaction with binding sites. Hox proteins with relatively high affinity for their preferred binding sites, and/or with the ability to effectively use multiple binding sites to increase affinity, could compete with chromatin to establish binding at the relatively closed chromatin environments of the selective sites. Hox proteins with high numbers of unique binding regions, e.g., Pb, Dfd, and Abd-B, would have a high affinity for their preferred sites. On the other hand, Hox proteins, such as Antp and Ubx, would have a lower affinity and be unable to reach the affinity threshold for effective competition with chromatin and so would be restricted to binding less selective open chromatin regions. This could be termed a quantitative affinity model. Alternatively, selectivity could be based on more qualitative differences between Hox proteins, for example, they could differ in their ability to bind to nucleosomal DNA, or in their ability to interact with other DNA-binding proteins with whom they could collaborate to compete with chromatin [8, 30–32]. A third possibility is that their differential ability to interact with relatively inaccessible chromatin could depend on selective ability to interact with chromatin remodelers to open chromatin at their binding sites.

We have investigated the influence of other DNA-binding proteins on Hox binding in chromatin in two different situations: provision of the canonical Hox cofactors Exd/Hth and provision of the pioneer factor Gcm. The Exd/Hth cofactors physically interact with Hox proteins through binding between Exd and the Hox YPWM motif and other interfaces [33–37]. Provision of Exd/Hth together with individual Hox proteins in Kc167 cells has clear effects on Hox binding, generally resulting in an increase in the number of significant Hox binding sites, promotion of chromatin opening, and a shift in the prior chromatin accessibility profile of bound sites towards less accessible chromatin. The Exd/Hth-enhanced Hox binding regions show little Hth binding or chromatin opening when Exd/Hth is expressed in the absence of Hox. This contrasts with evidence from vertebrate studies where Exd and Hth homologs, Pbx and Meis respectively, act as pioneer factors at specific sites. Pbx acts to initiate muscle development by marking specific genes for activation by MyoD [38]. Pbx and Meis collaborate to bind to an H1-compacted enhancer, recruiting PARP1 and leading to the PARP1-mediated eviction of H1 from chromatin [39]. At Hox binding sites, Pbx and Meis have been observed to precede Hox binding [40] although there is also collaboration as Hoxa2 binds to a set of Meis pre-bound sites leading to enhanced Meis binding [41]. In our genomic analysis, we find little support for the pioneer function of Hth but rather Hox and Exd/Hth appear to work together to open chromatin and promote Hox binding. Exd/Hth-enhanced Hox binding regions are strongly enriched in Exd-Hox consensus dimeric binding sites. Overall, the effects of Exd/Hth on Hox binding suggest Exd/Hth provides an increase in binding affinity at the Exd/Hth-enhanced Hox binding sites promoting enhanced competition with chromatin and raising the chromatin accessibility threshold for each Hox protein. The resulting general shift of the chromatin accessibility profile for each Hox protein towards less accessible chromatin fits with the quantitative affinity model. In the second situation, we provide Gcm, a protein that does not physically interact with Hox proteins [30] but which we show has the ability to open chromatin. We tested the effects of providing Gcm in conjunction with either Dfd or Ubx and found that chromatin opening by Gcm generated novel Hox binding sites but more for Ubx than Dfd, which fits with the ability of Dfd to bind less accessible regions on its own. In contrast to the situation with Exd/Hth, with Gcm, we see no evidence for collaborative effects on chromatin opening. Gcm presents an example of a DNA binding protein that alters the chromatin accessibility landscape thereby affecting Hox binding without necessarily having a direct physical interaction with Hox proteins. This may be a general way that other DNA-binding proteins influence Hox protein targeting through the strong effect of chromatin accessibility on Hox binding, with the almost complete occupancy of the most highly accessible regions (Fig. 10) indicating that binding is dependent on accessibility per se without the necessity for interactions with specific partner proteins.

Although Exd/Hth has a strong effect on the number and accessibility of Hox binding regions, we see the little general effect of Exd/Hth on the selectivity of Hox binding in vivo. However, particularly for Antp, Ubx, and Abd-B, the provision of Exd/Hth alters the Hox binding specificity as seen in the increased relative enrichment of the Abd-B motif (HoxB) versus the anterior Hox motifs (HoxA). This may occur through conformational constraints on Hox proteins in the Hox/Exd/Hth complex as with the phenomenon of latent specificity seen in vitro [16].

The specific case of the interaction of Abd-B with Exd/Hth is interesting since Abd-B lacks the YPWM motif, although it may interact with Exd through other interfaces [36], and its binding affinity for DNA in vitro is not increased by Exd [15]. In our data, Abd-B does not follow the same trend as the other Hox proteins in that provision of Exd/Hth does not increase the number of significant peaks detected by Abd-B ChIP. Furthermore, there is no general enhancement in binding to less accessible chromatin, since we observed no decrease in the average ATAC scores for Abd-B binding regions nor any change in the accessibility profile (Fig. 5 and Additional file 1: Figure S1). On the face of it, these observations suggest that Abd-B may not interact with Exd/Hth in vivo. However, further examination of differential binding reveals that there is in fact a significant set of regions where Abd-B binding is enhanced in the presence of Exd/Hth (351 regions at logFC 1 in the transient data set) and in these regions, Exd/Hth promotes Abd-B binding to less accessible chromatin (Fig. 5a, d). These regions show strong enrichment for Abd-B, Exd, and Hth motifs (Fig. 5f), and the dimeric Exd-Hox site TGATTTAT is the most enriched motif found by de novo motif analysis on the set of regions that show enhanced binding of Abd-B in the presence of Exd/Hth (Additional file 1: Figure S4). Thus, Abd-B may interact with Exd/Hth at a subset of sites in vivo. On the other hand, there is a significant set of regions that show decreased Abd-B binding in the presence of Exd/Hth (1648 regions at logFC 1 in the stable line data). Furthermore, analysis of ChIP scores in the transient transfection data (Additional file 1: Figure S7) shows that, particularly in closed chromatin, there are clear populations of peaks bound exclusively in the presence or absence of Exd/Hth. Examination of motif occurrence in these two populations shows that, as expected, the Exd/Hth-dependent peaks show the highest occurrence of ExdHox sites; however, the peaks exclusively present in the absence of Exd/Hox show a higher occurrence of Hox sites and particularly high numbers of sites/region. We interpret this as support for the importance of multiple binding sites in a binding region allowing Abd-B to access relatively closed chromatin and speculate that the presence of Exd/Hth may interfere with the multiple binding of Abd-B. This interaction fits with the antagonism between Abd-B and Exd/Hth described previously [42].

Our data reveal a clear relationship between Hox specificity and binding site affinity (Fig. 4). We find that the regions associated with highly selective Hox binding show high TBA, based on multiple binding sites with high scoring matches to Hox consensus binding sites. This fits with the observation that these sites are in less accessible chromatin, suggesting that high affinity is required for effective competition with chromatin. However, this relationship contrasts with the evidence from in vitro SELEX studies and observations at the *ovo/shavenbaby* locus in vivo where highly selective Hox binding is associated with low-affinity binding sites [43]. Interestingly, we find that, while Hox selectivity is linked to high-affinity sites over the whole set of binding regions, if we examine the relationship over the subset of regions with higher chromatin accessibility (ATAC scores > 25), the relationship is reversed so that higher selectivity is associated with lower TBA (Additional file 1: Figure S1a,b). Thus, the link between weak binding sites and high Hox selectivity may be applicable in highly open chromatin.

Several features of the binding data for the different Hox proteins, notably the fraction of unique sites (Fig. 1d) and the profiles of chromatin accessibility (Fig. 3a), show an intriguing graded relationship to the sequence of Hox gene expression along the anterior-posterior body axis. For both these features, the central Hox genes represent a minimum state; for example, the ability to access more closed chromatin progressively increases both anteriorly and posteriorly from a low point represented by Antp/Ubx (Fig. 3a). This graded relationship is reminiscent of the classic Hox phenomenon of posterior dominance whereby more posterior Hox genes over-rule the functions of anterior Hox genes [44–48]. However, although the chromatin accessibility profiles follow the sequence of posterior dominance running posteriorly from Antp to Abd-B, anteriorly the trend is reversed. It is interesting that, while the dominance relationships seem relatively straightforward from *Antp* posteriorly, the hierarchical relationships among the more anterior Hox genes are more complicated [49]. Although the functional hierarchy based on heat-shock induced over-expression of Hox proteins suggested an overall anterior-posterior sequence [46, 48], more recent experiments using the *nullo* promoter to drive early ubiquitous Hox expression point to a reversed hierarchy among the anterior Hox genes [50]. While ectopic expression of Hox genes from *Antp* posteriorly leads to posterior-wards transformations consistent with posterior dominance, ectopic expression of more anterior Hox genes leads to anterior-wards transformations, indicating a reversal of the dominance hierarchy. This interpretation is supported by loss-of-function phenotypes which, from *Scr* anteriorly give rise to posterior-wards transformation, and from *Antp* posteriorly result in anterior-wards transformations [50]. These relationships fit with the idea of an evolutionary and developmental ground state represented by the second thoracic segment or the Hox gene *Antp* [50, 51]. Linking this with the anterior-wards and posterior-wards graded relationships we see in the chromatin accessibility profiles, suggests that in building on the ground state the progressive ability of Hox proteins to engage with binding sites in less accessible chromatin may be a key feature of the evolutionary mechanism of segment diversification.

Overall, our studies indicate the role played by chromatin accessibility in Hox target selection and the observation that Hox binding is much more closely correlated with chromatin accessibility than with binding affinity has implications for other systems in understanding the relationship between genome sequence and transcription factor binding. It fits with studies on transcription factor binding in the *Drosophila* blastoderm [4, 52] and echoes the recent observation that interpretation of the gradient of the homeodomain protein Bicoid, in establishing the anterior-posterior axis in the *Drosophila* embryo, is more dependent on chromatin accessibility than on the binding affinity of target sites [53].

## Conclusions

Our studies reveal a strong link between chromatin accessibility and target selection by Hox proteins. In particular, we show that target sites with highly selective Hox binding have two key properties; they are relatively less accessible and they have relatively higher Hox protein binding affinity. This suggests that selective binding may depend on the ability of particular Hox proteins to use their higher binding affinity to compete with nucleosomes to access their specific targets. Other proteins binding close to Hox target sites play a role by establishing the chromatin accessibility landscape and we demonstrate the effect of the pioneer protein, Gcm, on Hox binding. The effects of the canonical Hox cofactors, Extradenticle and Homothorax, are also linked to chromatin accessibility. We find they strongly influence Hox binding by enabling access to target sites in less accessible chromatin. Although these cofactors have been proposed to facilitate selective Hox binding, we find they generally increase the number of Hox binding sites with little effect on overall Hox selectivity. In summary, our results provide a basis for understanding Hox selectivity, with competition between transcription factors and nucleosomes enabling small differences in binding specificities to be exploited to achieve target discrimination. We suggest that this mechanism is also likely to be relevant for achieving selective binding in other transcription factor families.

## Materials and Methods

### Cell culture

Kc167 cells (obtained from the Drosophila Genomics Resource Center) were cultured in Schneider’s medium supplemented with 5% fetal calf serum and antibiotics at 25°C.

### Expression plasmid cloning

Coding sequences (CDSs) for the eGFP-tagged Hox proteins Ubx, Abd-A, and Abd-B and for the Hth cofactor derived from Hox-vectors produced by Beh et al. [18]. CDSs for the remaining Hox proteins Lab, Pb, Dfd, Scr, and Antp and for Exd and Gcm were amplified from a cDNA preparation (QIAGEN, 205310) of 0–12-h-old embryos via nested PCR, starting with primers specific to flanking UTRs of each target CDS. All DNA amplifications were done using a Phusion High-Fidelity DNA Polymerase (NEB, M0530).

For transient transfection, sequences encoding eGFP-tagged transcription factors were cloned into the pMT expression vector (Invitrogen V4120-20), which employs the inducible *Drosophila* metallothionein promoter to drive transgene expression using a suitable CuSO_4_ concentration in the growing medium.

To generate stable Kc167 cell lines carrying inducible eGFP-tagged factors (stable lines), CDSs were cloned into the pMT-puro expression vector (Addgene, #17923), which uses a puromycin selection system. We produced stable lines by selecting cells in medium with 5 μg/ml of puromycin after transfection with pMT-puro-Hox constructs (see below).

We produced vectors expressing either single eGFP-tagged Hox proteins, Hth and Gcm (monocistronic vectors), or eGFP-tagged Hox factors in association with Hth, eGFP-Exd in association with Hth, and specific Hox factors in association with Gcm (bicistronic vectors). We employed the T2A peptide self-cleavage system for multicistronic constructs. All constructs were sequence-verified.

### Transfection

Transient transfection was performed according to Beh et al. [18]. Briefly, Kc167 cells harvested in log phase were used to seed 10 cm dishes (Corning Inc. 353003) at a density of 2.5 × 10^7^ cells per dish and transfection was performed using FuGENE 6 Transfection Reagent (Promega E2691) according to the manufacturer’s instructions. Dishes were then incubated at 25°C for approximately 14 h. For stable transfection, 2 × 10^6^ cells were re-suspended in 10 ml Schneider’s medium containing the transfection solution (70 μl OPTIMEM, 3 μl Fugene, and 2 μg plasmid DNA) and seeded into 10 cm dishes. After incubation at 25°C for approximately 18 h, the medium was replaced with standard Schneider’s and cells were cultured for approximately 24 h before starting puromycin selection.

### Induction of gene expression, fixation, and FACS

For transiently transfected cells, the medium was replaced by 10 ml of Schneider’s medium/1 mM CuSO_4_ and dishes were incubated at 25°C for 4 h to induce Hox-GFP expression. In the case of stable lines, CuSO_4_ concentrations and induction times varied between lines and were adjusted to provide optimal expression levels prior to FACS sorting. Cell fixation and FACS sorting methods were as described in Beh et al. [18]. Cells destined for ATAC experiments were not fixed and were FACS sorted into PBS, 0.1% BSA instead of PBS, and 0.01% Triton X-100. Sorting was performed using a MoFlo FACS machine (Beckman Coulter) equipped with a 488-nm argon laser (100 mW). For each sort, the flow cytometer was calibrated with AcGFP Flow Cytometer Calibration Beads (Clontech #632594) and cells were sorted by gating in the same fluorescence intensity range. The range was set based on the Ubx-GFP profile and, based on comparison with the calibration beads, corresponds to a range of 38,000 to 74,000 Hox-GFP molecules per cell [18]. This allowed us to sort each time a population of GPF-positive cells expressing an equal range of Hox-GFP molecules in the physiological range.

An equal number of cells (10^6^) was sorted for all samples.

### ChIP and ChIP-seq library preparation

ChIP was performed as in Beh et al. [18] except the anti-GFP antibody used in this study was from Sigma (G1544; 2 μl per ChIP). ChIP and input DNA were re-suspended in 20 μl of TE buffer. Ten microliters of ChIP DNAs and 400 pg of input DNA in 10 μl TE buffer were used to produce sequencing libraries using the SMARTer ThruPLEX DNA-seq Kit (Takara Bio Inc.) in accordance with the sample preparation guide. Fourteen cycles of amplification were used for all libraries.

### ATAC-seq

ATAC-seq libraries were prepared according to Buenrostro et al. [54]. Final libraries were size selected to contain molecules of 150–700 bp using AMPure XP beads (Beckman Coulter).

### Sequencing and data processing

Libraries were either sequenced on the Illumina HiSeq 2000 or HiSeq 4000 platforms at the CRUK Cambridge Institute Genomics Core. ChIP-seq and ATAC-seq reads were aligned to the *Drosophila melanogaster* genome release 6 (dm6) excluding scaffolds using bowtie (v 1.2.2) with the -m1 option. Reads were then converted to bam files with Samtools (v 1.3.1). ChIP-seq peak detection for each biological replicate using the input as background was performed with MACS2 (v 2.1.1.20160309) using --keep-dup 1, --call-summits, and -q 1e−2 and -q 1e−10 options. Binding regions overlapping exon regions contained in the plasmid were then removed. Bound regions were defined as the union of overlapping regions detected by MACS2 across both replicates at a given stringency. Unless stated otherwise, we use *q* value 1e−2 in the figure plots.

ATAC-seq reads aligning to the + strand were offset by +4 bp and reads aligning to the - strand were offset −5 bp to represent the center of the transposase binding, then the reads were extended by 5 bp on either side. Open regions for the basal Kc cells were then called using MACS2 with options --shift -45 --extsize 100 and -q 1e-2 for each of the 3 replicates. Basal ATAC core “open” regions were defined as the union of open regions present in at least 2 of the replicates and regions not defined as “open” were called “closed” (see Additional file 1: Tables S1-3 for the ChIP-Seq read overview, ChIP-Seq binding region numbers, and ATAC-seq read overview.

### Hox group peak regions

To define bound regions between all Hox and Hox+Cofactor(s) ChIP samples, the sub-peak summit positions at MACS *q* value 1e−10 were grouped using the GenomicRanges R package [55]. Starting with the sample with the largest number of sub-peak summits, these summit positions were extended ± 100 bp and then overlapped with the extended summits of the next sample. A new center position was then calculated using the mean position between all sub-peak summits belonging in this grouped region. All non-overlapping summit positions were taken to the next round. Finally, group regions containing less than 2 members were removed. This resulted in 200 bp peak regions. For the transiently transfected data which includes all 8 Hox and Hox+Hth samples, this resulted in 15,945 regions, called Hox group peaks. For the stable cell line data, we used the 3 Hox samples (Dfd, Ubx, and Abd-B) and Hox+cofactors (Hth and Gcm) samples which resulted in 21,002 regions now called Hox group peaks (stable). Each Hox group peak region was then flagged as bound by a specific Hox (or Hox+cofactor) if peak regions of both replicates at the selected stringency overlapped (we used min overlap 1 bp throughout). Additionally, the regions were flagged as open if they overlapped with the Kc cell basal core open regions. The Hox group peak regions for the transiently transfected data are detailed in Additional file 2: Table S6.

### Cofactor-enhanced binding analysis

Reads overlapping the Hox group peaks were counted using the union method of the summarize overlaps function in the GenomicAlignments R package by extending the reads by their fragment size (as determined by MACS2). The count table was then processed with R package edgeR [56] as follows: reads were normalized using the loess method (as per the csaw R package; [57]) to remove trended bias, then the dispersions were calculated and the glmQLFit function used to fit a quasi-likelihood negative binomial generalized log-linear model to count data. Differential binding (DB) analysis was performed per pair-wise comparison between two samples using a threshold of fdr ≤ 0.01 and logFC ≥1 (in this case log difference of binding signal); additionally, both replicates of the DB sample were required to be bound at macs *q* value 1e−2 (Additional file 1: Table S4).

### ChIP and ATAC scores

The ChIP-seq reads of both replicates were extended to match the mean fragment size. ATAC-seq reads of both replicates were extended by 100 bp centered on the Tn5 cut site. Bedgraph files were then created using MACS2 pileup and scaled to reads-per-million, counting reads overlapping the Hox group peaks for each experiment. The profiles were then binned at 20 bp resolution using the mean score. ChIP or ATAC scores of selected regions were then calculated as the mean profile score of overlapping bins.

### Venn diagrams

The highest binding score position in regions bound by both replicates at the selected stringency was extended by ± 200 bp. To deal with the problem of one region overlapping with two (or more) regions in the other sample, we created the union of these regions across the three Hox samples under investigation, thus creating a unique region set. For each individual Hox, the overlap with the union region was quantified and plotted as a proportion sized Venn diagram using the eulerr R package [58].

### Motif analysis

Motif enrichment analysis was performed using the R package PWMEnrich with the motifs from the MotifDb database [59]. The motif numbers per Hox protein are Lab 2, Pb 3, Dfd 4, Scr 3, Antp 4, Ubx 3, Abd-A 3, and Abd-B 4. For Exd, we used 3 motifs, excluding the exd_FlyReg_FBgn0000611 motif as an outlier and we used 4 motifs for Hth. Motif enrichment scores [log_10_(1/*p* value)] were grouped by transcription factor and individual motifs plotted as dot plots with the median as colored bar or grouped into HoxA* (Lab, Pb, Dfd, Scr, Antp, Ubx, Abd-A) and Abd-B and plotted as boxplots using R. For total binding affinity (TBA) analysis [24], the Hox PWMs (truncated to 7-mers) from the JASPAR database were used and we combined the PWMs of Lab, Pb, Dfd, Scr, Antp, Ubx, and Abd-A to a new PWM HoxA and renamed Abd-B to HoxB (Fig. 3b). For the TBA analysis on individual Hox motifs (in Fig. 4), we used the truncated JASPAR Hox PWMs. TBA was calculated across the 200 bp Hox group peaks (Fig. 3e, Fig. 4d, e, Additional file 1: Figure S1a,b) or 200 bp binding summit regions (summit position extended by ± 100 bp; Fig. 3c) using the MatrixRider R package [60]. For Hox site counting, sequences were searched using the truncated Hox 7-mer JASPAR PWMs with the Biostrings R package [61], matchPWM function with min.score = 80% on both strands and all possible sites (allowing overlaps) counted. For max PWM score, the highest score within each sequence for each PWM was extracted (using min.score ≥ 50%).

De-novo motif discovery was performed using HOMER [62] on the cofactor-enhanced binding regions (Hox+Hth) (Additional file 1: Table S4, Additional file 1: Figure S4). All sequence logos were plotted using the seqLogo R package [63]*.*

Consensus matrixes for Fig. 7a were created with the Biostrings R package finding all matches to TGATTDAT (where D = A or G or T), based on the in vitro SELEX Exd-Hox sites [16] and our HOMER de-novo motifs, allowing 1 mismatch in cofactor-enhanced binding regions. The binding regions used were class 1 unique Exd/Hth enhanced Lab bound, class 2 unique Exd/Hth enhanced Pb or Dfd or Scr bound, and class 3 unique Exd/Hth enhanced Antp or Ubx or Abd-A or Abd-B bound.

The top 15 prevalent 8-mer sequence patterns in Exd/Hth-enhanced binding regions were determined using Biostrings, masking identified *k*-mers after each round.

SELEX raw data was downloaded from GSE65073 [64] and reprocessed using the SELEX R package [65] with optimal length = 12 and Markov order = 5, to obtain complete affinity tables for each Exd-Hox experiment. The affinities for the three Exd-Hox class patterns in Fig. 7a were then looked up locating any 12-mer containing these patterns (or the reverse complement) and plotted as a stripchart plot using R.

### Correlation heatmap

The union of all regions bound by Hox or Hox+Hth at MACS *q* value 1e-2 was tiled into 20 bp windows and reads overlapping each window were counted using the csaw R package [57]. Reads were then normalized by library size and transformed to counts per million. The correlation between the samples was then plotted using heatmap.2 from the gplots R package.

### Chromatin accessibility analysis

The 10 bp adjusted ATAC-seq reads overlapping the 21,002 Hox group peaks (stable) regions were counted as above. These counts were then processed as for the cofactor-enhanced binding analysis. We defined significantly increased chromatin accessibility regions as edgeR fdr ≤ 0.01 and logFC ≥ 1.5 comparing induced versus non-induced samples (Additional file 1: Table S5).

### Occupancy heatmaps in open regions

Open chromatin regions in basal Kc cells (16,118 regions ranging in size between 100–2413 bp) were tiled into 200 bp bins as follows: smaller regions were resized to 200 bp fixed on the center of each region and larger regions were split into 200 bp tiles. The tiles were then classified as bound or not bound if they overlapped a Hox bound region. Mean ATAC scores of basal Kc cells and TBA for HoxA or HoxB PWMs were calculated per tile. The log of these scores was then linearly binned into 40 bins, and a heatmap plotted. For the “All regions” plot, the heatmap colors show the location of the highest density of these tiles. The colors in the other plots represent the proportion of Hox bound within each bin. We then assessed the correlation *R*^2^ of occupancy (proportion bound per bin) with chromatin accessibility (ATAC scores) or binding affinity (TBA), shown as scatterplots.

## Additional files

Additional file 1: **Figure S1.** The association between Hox specificity and binding site affinity, binding region numbers and chromatin accessibility profiles; **Figure S2.** Hox, Hox+Hth, Hth and Exd binding in open and closed chromatin; **Figure S3.** Motif analysis showing individual motifs; **Figure S4.** De-novo motif analysis of Exd/Hth cofactor enhanced binding regions; **Figure S5.** Comparing the effects of Exd/Hth and Gcm: Chromatin accessibility in Hox + Exd/Hth compared to Hox + Gcm; **Figure S6.** Hox occupancy is more strongly associated with binding region chromatin accessibility than with binding affinity; **Figure S7.** The presence of Exd/Hth leads to both enhanced and reduced Abd-B binding; **Figure S8.** Comparison of Ubx and Dfd binding in presence of Gcm for regions in basal Kc167 closed chromatin; **Table S1.** ChIP-seq read overview; **Table S2.** ChIP-Seq binding region numbers; **Table S3.** Stable cell lines ATAC-seq read overview; **Table S4.** Cofactor-enhanced binding analysis of transient data in Hox group peak regions; **Table S5.** Increased chromatin accessibility analysis. (PDF 5942 kb)

Additional file 2: **Table S6.** Hox group peak regions. (XLSX 3054 kb)

## Data Availability

The ChIP-Seq and ATAC-Seq data are available from GEO under accession number GSE122575 [66].

## References

[1] Jolma A, Yan J, Whitington T, Toivonen J, Nitta KR, Rastas P (2013). DNA-binding specificities of human transcription factors. Cell..

[2] Badis G, Berger MF, Philippakis AA, Talukder S, Gehrke AR, Jaeger SA (2009). Diversity and complexity in DNA recognition by transcription factors. Science..

[3] Noyes MB, Christensen RG, Wakabayashi A, Stormo GD, Brodsky MH, Wolfe SA (2008). Analysis of homeodomain specificities allows the family-wide prediction of preferred recognition sites. Cell..

[4] Kaplan T, Li XY, Sabo PJ, Thomas S, Stamatoyannopoulos JA, Biggin MD (2011). Quantitative models of the mechanisms that control genome-wide patterns of transcription factor binding during early Drosophila development. PLoS Genet..

[5] Pearson JC, Lemons D, McGinnis W (2005). Modulating Hox gene functions during animal body patterning. Nat Rev Genet..

[6] Hueber SD, Lohmann I (2008). Shaping segments: Hox gene function in the genomic age. BioEssays News Rev Mol Cell Dev Biol..

[7] Rezsohazy R, Saurin AJ, Maurel-Zaffran C, Graba Y (2015). Cellular and molecular insights into Hox protein action. Development..

[8] Mann RS, Lelli KM, Joshi R (2009). Hox specificity unique roles for cofactors and collaborators. Curr Top Dev Biol..

[9] Jacobs Y, Schnabel CA, Cleary ML (1999). Trimeric association of Hox and TALE homeodomain proteins mediates Hoxb2 hindbrain enhancer activity. Mol Cell Biol..

[10] Ryoo HD, Marty T, Casares F, Affolter M, Mann RS (1999). Regulation of Hox target genes by a DNA bound Homothorax/Hox/Extradenticle complex. Dev Camb Engl..

[11] Shen WF, Rozenfeld S, Kwong A, Köm ves LG, Lawrence HJ, Largman C (1999). HOXA9 forms triple complexes with PBX2 and MEIS1 in myeloid cells. Mol Cell Biol..

[12] Longobardi E, Penkov D, Mateos D, De Florian G, Torres M, Blasi F (2014). Biochemistry of the tale transcription factors PREP, MEIS, and PBX in vertebrates. Dev Dyn Off Publ Am Assoc Anat..

[13] Chan SK, Jaffe L, Capovilla M, Botas J, Mann RS (1994). The DNA binding specificity of Ultrabithorax is modulated by cooperative interactions with extradenticle, another homeoprotein. Cell..

[14] Chang CP, Shen WF, Rozenfeld S, Lawrence HJ, Largman C, Cleary ML (1995). Pbx proteins display hexapeptide-dependent cooperative DNA binding with a subset of Hox proteins. Genes Dev..

[15] van Dijk MA, Murre C (1994). extradenticle raises the DNA binding specificity of homeotic selector gene products. Cell..

[16] Slattery M, Riley T, Liu P, Abe N, Gomez-Alcala P, Dror I (2011). Cofactor binding evokes latent differences in DNA binding specificity between Hox proteins. Cell..

[17] Galant R, Walsh CM, Carroll SB (2002). Hox repression of a target gene: extradenticle-independent, additive action through multiple monomer binding sites. Dev Camb Engl..

[18] Beh CY, El-Sharnouby S, Chatzipli A, Russell S, Choo SW, White R (2016). Roles of cofactors and chromatin accessibility in Hox protein target specificity. Epigenetics Chromatin..

[19] Biggin MD (2011). Animal transcription networks as highly connected, quantitative continua. Dev Cell..

[20] Krause HM, Klemenz R, Gehring WJ (1988). Expression, modification, and localization of the fushi tarazu protein in Drosophila embryos. Genes Dev..

[21] Little SC, Tkačik G, Kneeland TB, Wieschaus EF, Gregor T (2011). The formation of the Bicoid morphogen gradient requires protein movement from anteriorly localized mRNA. PLoS Biol..

[22] Walter J, Dever CA, Biggin MD (1994). Two homeo domain proteins bind with similar specificity to a wide range of DNA sites in Drosophila embryos. Genes Dev..

[23] Foat BC, Morozov AV, Bussemaker HJ (2006). Statistical mechanical modeling of genome-wide transcription factor occupancy data by MatrixREDUCE. Bioinforma Oxf Engl..

[24] Grassi E, Zapparoli E, Molineris I, Provero P (2015). Total binding affinity profiles of regulatory regions predict transcription factor binding and gene expression in human cells. PloS One..

[25] Bazzi W, Cattenoz PB, Delaporte C, Dasari V, Sakr R, Yuasa Y, et al. Embryonic hematopoiesis modulates the inflammatory response and larval hematopoiesis in Drosophila. eLife. 2018;7:e34890. 10.7554/eLife.34890 PMC6040882 29992900

[26] Bernardoni R, Vivancos V, Giangrande A (1997). glide/gcm is expressed and required in the scavenger cell lineage. Dev Biol..

[27] Cherbas L, Willingham A, Zhang D, Yang L, Zou Y, Eads BD (2011). The transcriptional diversity of 25 Drosophila cell lines. Genome Res..

[28] Beachy PA, Varkey J, Young KE, von Kessler DP, Sun BI, Ekker SC (1993). Cooperative binding of an Ultrabithorax homeodomain protein to nearby and distant DNA sites. Mol Cell Biol..

[29] Desplan C, Theis J, O’Farrell PH (1988). The sequence specificity of homeodomain-DNA interaction. Cell..

[30] Sorge S, Ha N, Polychronidou M, Friedrich J, Bezdan D, Kaspar P (2015). The cis-regulatory code of Hox function in Drosophila. EMBO J..

[31] Baëza M, Viala S, Heim M, Dard A, Hudry B, Duffraisse M, et al. Inhibitory activities of short linear motifs underlie Hox interactome specificity in vivo. eLife. 2015;4:06034. 10.7554/eLife.06034 PMC4392834 25869471

[32] Zouaz A, Auradkar A, Delfini MC, Macchi M, Barthez M, Ela Akoa S (2017). The Hox proteins Ubx and AbdA collaborate with the transcription pausing factor M1BP to regulate gene transcription. EMBO J..

[33] Knoepfler PS, Kamps MP (1995). The pentapeptide motif of Hox proteins is required for cooperative DNA binding with Pbx1, physically contacts Pbx1, and enhances DNA binding by Pbx1. Mol Cell Biol..

[34] Phelan ML, Rambaldi I, Featherstone MS (1995). Cooperative interactions between HOX and PBX proteins mediated by a conserved peptide motif. Mol Cell Biol..

[35] Neuteboom ST, Peltenburg LT, van Dijk MA, Murre C (1995). The hexapeptide LFPWMR in Hoxb-8 is required for cooperative DNA binding with Pbx1 and Pbx2 proteins. Proc Natl Acad Sci U S A..

[36] Dard A, Reboulet J, Jia Y, Bleicher F, Duffraisse M, Vanaker J-M (2018). Human HOX proteins use diverse and context-dependent motifs to interact with TALE class cofactors. Cell Rep..

[37] Foos N, Maurel-Zaffran C, Maté MJ, Vincentelli R, Hainaut M, Berenger H (2015). A flexible extension of the Drosophila ultrabithorax homeodomain defines a novel Hox/PBC interaction mode. Struct Lond Engl 1993.

[38] Berkes CA, Bergstrom DA, Penn BH, Seaver KJ, Knoepfler PS, Tapscott SJ (2004). Pbx marks genes for activation by MyoD indicating a role for a homeodomain protein in establishing myogenic potential. Mol Cell..

[39] Hau A-C, Grebbin BM, Agoston Z, Anders-Maurer M, Müller T, Groß A (2017). MEIS homeodomain proteins facilitate PARP1/ARTD1-mediated eviction of histone H1. J Cell Biol..

[40] Choe S-K, Ladam F, Sagerström CG (2014). TALE factors poise promoters for activation by Hox proteins. Dev Cell..

[41] Amin S, Donaldson IJ, Zannino DA, Hensman J, Rattray M, Losa M (2015). Hoxa2 selectively enhances Meis binding to change a branchial arch ground state. Dev Cell..

[42] Rivas ML, Espinosa-Vázquez JM, Sambrani N, Greig S, Merabet S, Graba Y, et al. Antagonism versus cooperativity with TALE Cofactors at the base of the functional diversification of Hox protein function. PLoS Genet. 2013;9:e1003252. 10.1371/journal.pgen.1003252 PMC3567137 23408901

[43] Crocker J, Abe N, Rinaldi L, McGregor AP, Frankel N, Wang S (2015). Low affinity binding site clusters confer hox specificity and regulatory robustness. Cell..

[44] Hafen E, Levine M, Gehring WJ (1984). Regulation of Antennapedia transcript distribution by the bithorax complex in Drosophila. Nature..

[45] Struhl G, White RA (1985). Regulation of the Ultrabithorax gene of Drosophila by other bithorax complex genes. Cell..

[46] González-Reyes A, Morata G (1990). The developmental effect of overexpressing a Ubx product in Drosophila embryos is dependent on its interactions with other homeotic products. Cell..

[47] Struhl G (1983). Role of the esc+ gene product in ensuring the selective expression of segment-specific homeotic genes in Drosophila. J Embryo Exp Morph..

[48] González-Reyes A, Urquia N, Gehring WJ, Struhl G, Morata G (1990). Are cross-regulatory interactions between homeotic genes functionally significant?. Nature..

[49] Duboule D, Morata G (1994). Colinearity and functional hierarchy among genes of the homeotic complexes. Trends Genet..

[50] Gehring WJ, Kloter U, Suga H (2009). Evolution of the Hox gene complex from an evolutionary ground state. Curr Top Dev Biol..

[51] Lewis EB (1978). A gene complex controlling segmentation in Drosophila. Nature..

[52] Li X-Y, Thomas S, Sabo PJ, Eisen MB, Stamatoyannopoulos JA, Biggin MD (2011). The role of chromatin accessibility in directing the widespread, overlapping patterns of Drosophila transcription factor binding. Genome Biol..

[53] Hannon CE, Blythe SA, Wieschaus EF. Concentration dependent chromatin states induced by the bicoid morphogen gradient. eLife. 2017;6:e28275. 10.7554/eLife.28275 PMC5624782 28891464

[54] Buenrostro JD, Wu B, Chang HY, Greenleaf WJ (2015). ATAC-seq: a method for assaying chromatin accessibility genome-wide. Curr Protoc Mol Biol..

[55] Lawrence M, Huber W, Pagès H, Aboyoun P, Carlson M, Gentleman R (2013). Software for computing and annotating genomic ranges. PLoS Comput Biol..

[56] Robinson MD, McCarthy DJ, Smyth GK (2010). edgeR: a Bioconductor package for differential expression analysis of digital gene expression data. Bioinforma Oxf Engl..

[57] Lun ATL, Smyth GK (2016). csaw: a Bioconductor package for differential binding analysis of ChIP-seq data using sliding windows. Nucleic Acids Res..

[58] eulerr LJ (2018). Area-Proportional Euler and Venn Diagrams with Ellipses.

[59] Stojnic R, Diez D (2013). PWMEnrich: PWM enrichment analysis. R Package Version 262.

[60] MatrixRider GE (2015). Obtain total affinity and occupancies for binding site matrices on a given sequence.

[61] Pagès H, Aboyoun P, Gentleman R, DebRoy S (2018). Biostrings: Efficient manipulation of biological strings.

[62] Heinz S, Benner C, Spann N, Bertolino E, Lin YC, Laslo P (2010). Simple combinations of lineage-determining transcription factors prime cis-regulatory elements required for macrophage and B cell identities. Mol Cell..

[63] seqLogo BO (2018). Sequence logos for DNA sequence alignments.

[64] Abe N, Dror I, Yang L, Slattery M, Zhou T, Bussemaker HJ (2015). Deconvolving the recognition of DNA shape from sequence. Cell..

[65] Rastogi C, Liu D, Bussemaker H (2015). SELEX: Functions for analyzing SELEX-seq data.

[66] Porcelli D, Fischer B, Russell S, White R (2019). Chromatin accessibility plays a key role in selective targeting of Hox proteins. Datasets Gene expression omnibus.

