# Supplementary material for: Chromatin accessibility plays a key role in selective targeting of Hox proteins
Source: Genome Biol. 2019 Jun 3;20:115. doi: 10.1186/s13059-019-1721-4 (PMC6547607; doi:10.1186/s13059-019-1721-4)
Supplement: Supplementary file 1 — Figure S1. The association between Hox specificity and binding site affinity, binding region numbers and chromatin accessibility profiles; Figure S2. Hox, Hox+Hth, Hth and Exd binding in open and closed chromatin; Figure S3. Motif analysis showing individual motifs; Figure S4. De-novo motif analysis of Exd/Hth cofactor enhanced binding regions; Figure S5. Comparing the effects of Exd/Hth and Gcm: Chromatin accessibility in Hox + Exd/Hth compared to Hox + Gcm; Figure S6. Hox occupancy is more strongly associated with binding region chromatin accessibility than with binding affinity; Figure S7. The presence of Exd/Hth leads to both enhanced and reduced Abd-B binding; Figure S8. Comparison of Ubx and Dfd binding in presence of Gcm for regions in basal Kc167 closed chromatin; Table S1. ChIP-seq read overview; Table S2. ChIP-Seq binding region numbers; Table S3. Stable cell lines ATAC-seq read overview; Table S4. Cofactor-enhanced binding analysis of transient data in Hox group peak regions; Table S5. Increased chromatin accessibility analysis. (PDF 5942 kb) [file 13059_2019_1721_MOESM1_ESM.pdf]

Supplementary Figures

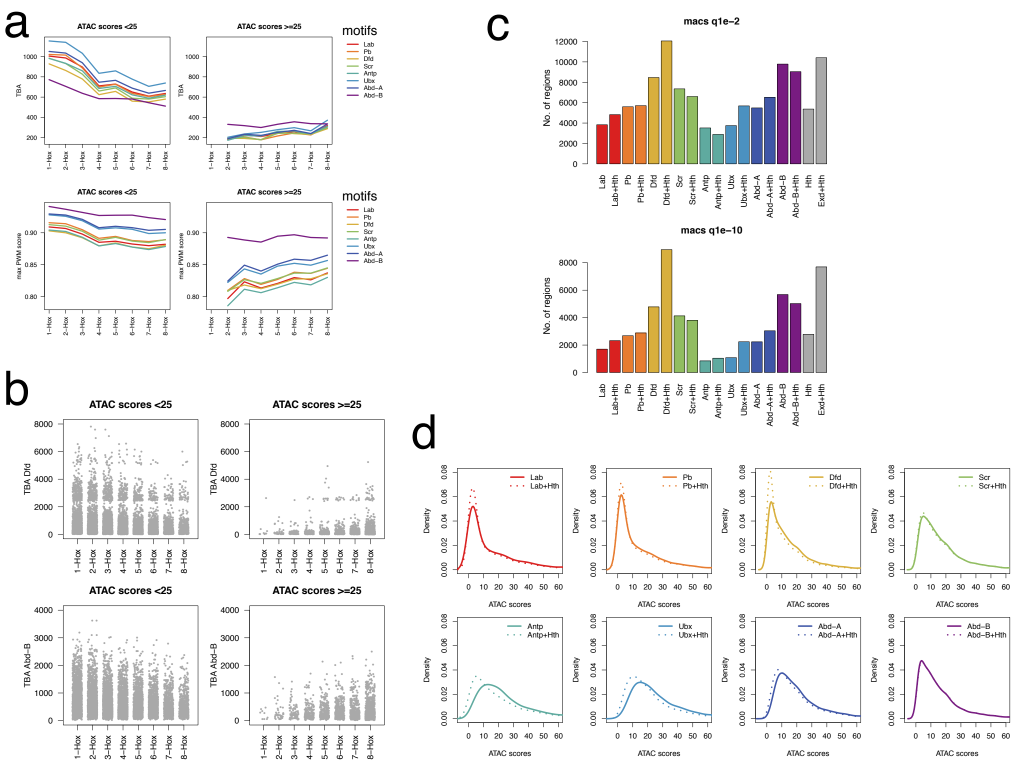

Figure S1 The association between Hox specificity and binding site affinity, binding region numbers and chromatin accessibility profiles.
a: Plots showing relationship between Hox selectivity and either (top row) TBA or (bottom row) highest PWM score for binding regions as in Figure 4a, but separated according to chromatin accessibility with more closed on left (ATAC scores < 25) and more open on right (ATAC scores >= 25). TBA is shown for each JASPAR 7-mer Hox PWM. The maxPWM score is the mean of the highest PWM score per region. Whilst the more closed regions show a positive association between Hox selectivity and both TBA and maxPWM score, the trend is reversed for the more open regions. Number of regions with ATAC scores < 25 is 13507 and number of regions with ATAC scores >= 25 is 2448. Data for 1-Hox is omitted due to low number of regions in this bin. b: TBA data for Dfd and Abd-B PWMs as in (a) plotted as strip plots showing clear reversal of association between TBA and Hox selectivity in more open versus less open chromatin. c: Numbers of regions bound by Hox and Hox+Hth for both replicates at q-value 1e-2 (upper) and higher stringency q-value 1e-10 (lower). d: Density plots of mean ATAC-seq scores for regions bound by Hox proteins with and without Exd/Hth showing the effect of the cofactors on the chromatin accessibility profile. Solid lines: Hox alone, dotted lines: Hox in presence of Exd/Hth.

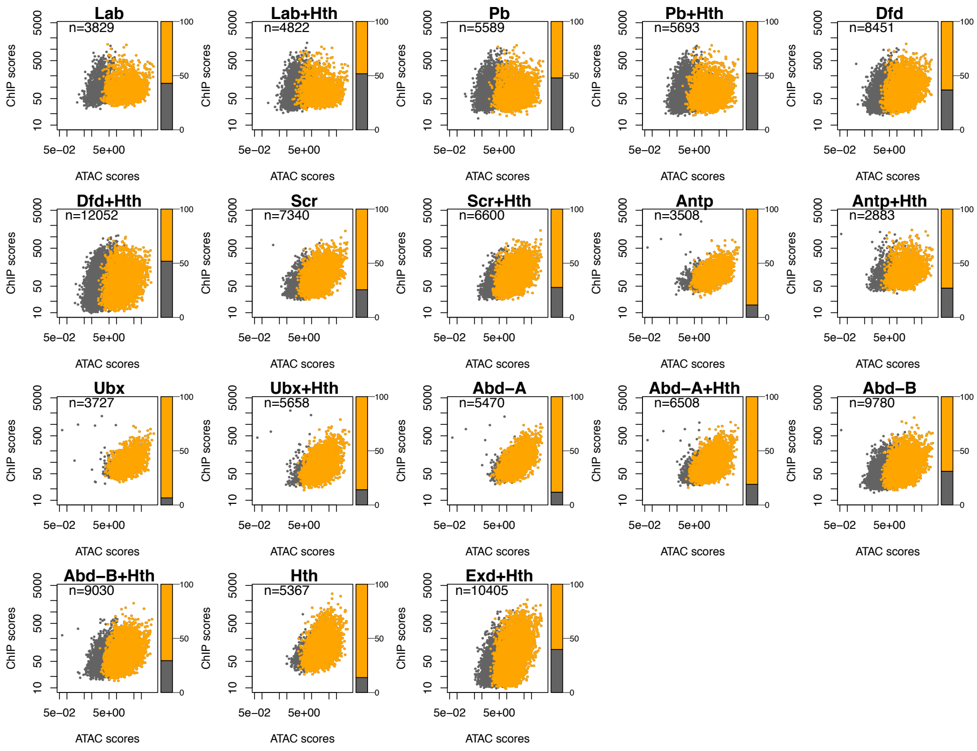

Figure S2 Hox, Hox+Hth, Hth and Exd binding in open and closed chromatin.
Scatter plots of ChIP score versus chromatin accessibility for each Hox and Hox+Hth ChIP and for Hth ChIP and ChIP of Exd in the presence of Hth. The binding summits positions bound at q-value 1e-10 were extended +/- 100bp and the mean of the ChIP scores and the mean of the basal Kc-cell ATAC scores plotted. Regions which overlap with open chromatin regions as determined using the same ATAC-seq data using macs2 at q1e-2 are shown in orange, grey regions indicate closed chromatin, i.e. regions do not overlap chromatin called as open. The barplot at the side indicate the proportion of closed and open regions. The plots show the different degrees of binding to closed chromatin for different Hox proteins and the general increase in binding to closed chromatin in the presence of Exd/Hth. Hth shows little binding to closed chromatin. Exd apparently shows more binding in closed chromatin, but note that much of this effect is due to the inclusion of lower ChIP scoring regions in the Exd+Hth plot. This is a result of lower scoring peaks reaching significance due to the low background in this ChIP.

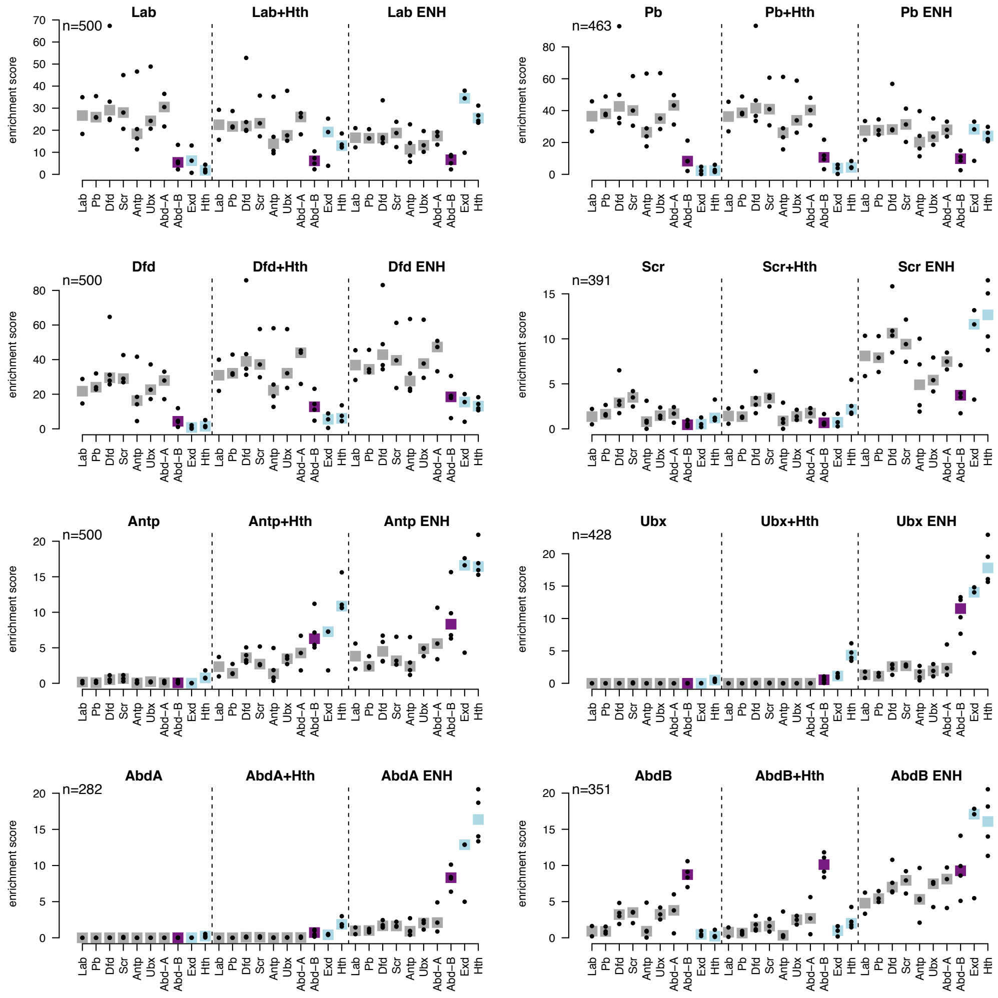

Figure S3 Motif analysis showing individual motifs.

Motif analysis comparing motif enrichment for Hox group regions for Hox alone (Hox), Hox in the presence of Exd/Hth (Hox+Hth) and Exd/Hth cofactor enhanced binding regions (Hox ENH) using the top (highest ChIP score) 500 regions from each class (or matched numbers to the ENH set where less than 500). Plot titles indicate binding region set used and motifs are indicated on the x-axis. Enrichment analysis was performed using PWMEnrich for the Hox motifs in the MotifDb database. Enrichment scores [$\log_{10}$(1/p-value)] for individual motifs are indicated (dots) together with the median for each motif set (bar). Lab, Pb, Dfd, Scr, Antp, Ubx and Abd-A (grey bar), Abd-B (purple bar), and the Exd and Hth motifs (light blue). Note the differences in Y-axis scale. Generally the provision of Exd/Hth has little effect on the Hox motif enrichments although the Exd and Hth motifs show increased enrichment. For Antp, Ubx and Abd-A the provision of Exd/Hth increases the relative enrichment of the Abd-B motif relative to the other Hox motifs, which may reflect the in vitro defined phenomenon of latent specificity [16].

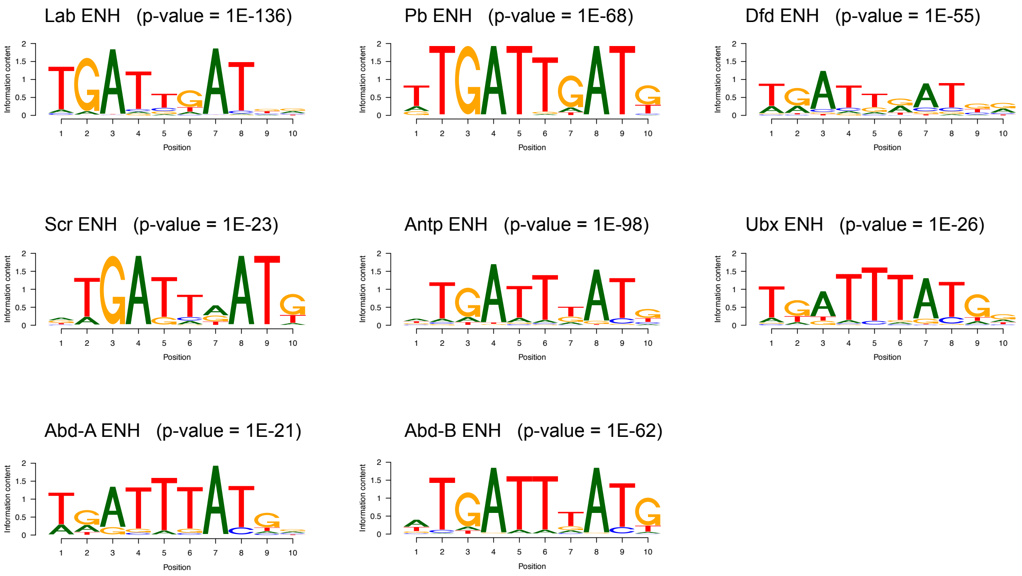

Figure S4 De-novo motif analysis of Exd/Hth cofactor enhanced binding regions.

PWMs for enriched motifs in Exd/Hth cofactor enhanced binding regions (Hox ENH) using HOMER. Enrichment p-values are shown.

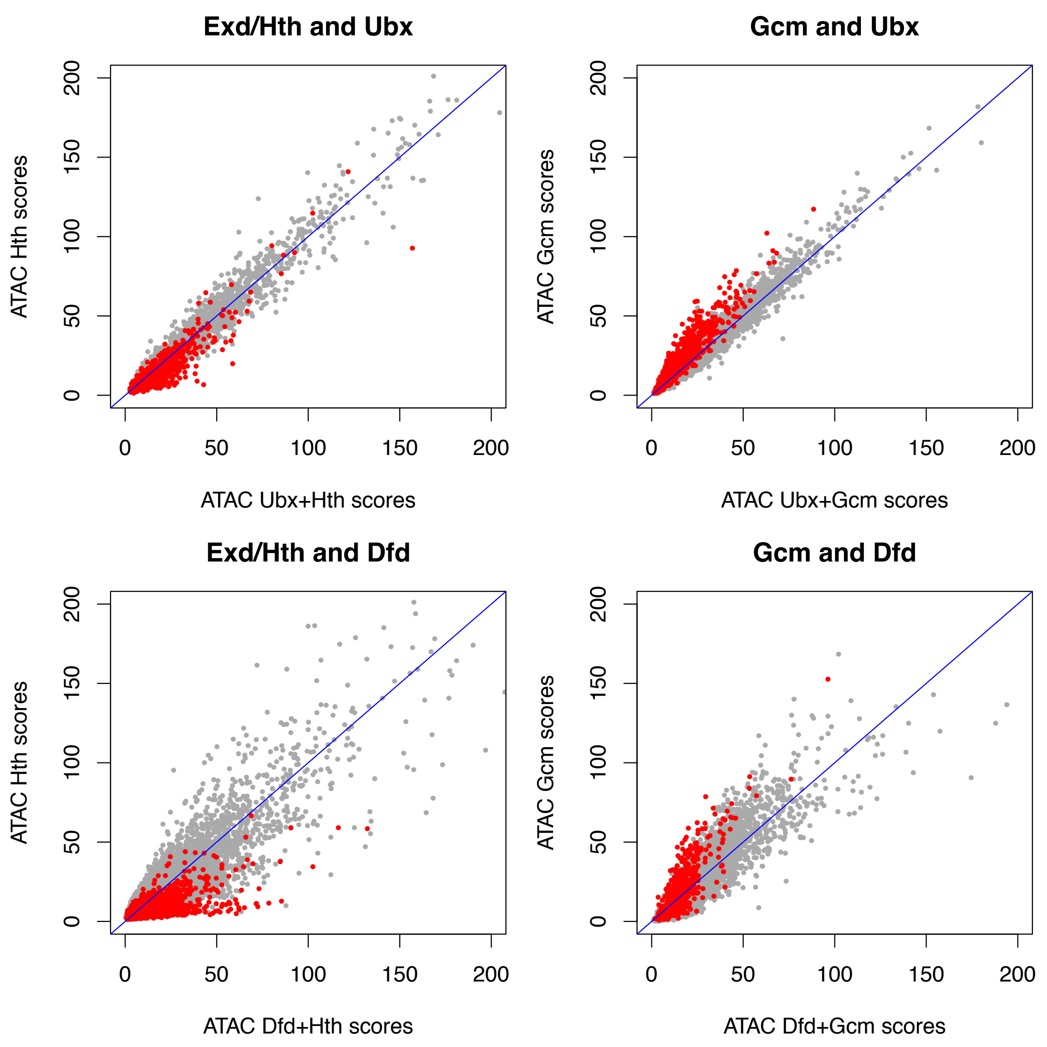

Figure S5 Comparing the effects of Exd/Hth and Gcm: Chromatin accessibility in Hox + Exd/Hth compared to Hox + Gcm.
Scatterplots comparing the effect on chromatin accessibility of providing Exd/Hth versus Gcm in addition to Hox proteins (Ubx or Dfd). Mean ATAC score per binding region are plotted for all the bound regions in Hox+Hth or Hox+Gcm respectively. On the Y-axis the ATAC scores are plotted for these regions in Hth or Gcm alone and on the X-axis the ATAC scores are plotted with the addition of Hox proteins (Ubx or Dfd). The Exd/Hth-enhanced or Gcm-enhanced regions respectively are shown in red. In the case of Exd/Hth, the Exd/Hth-enhanced Hox binding regions predominantly lie below the diagonal; i.e. they have increased chromatin accessibility in Hox+Hth than in Hth alone. This is not the case for Gcm. This illustrates the difference in the relationship between Hox proteins and Exd/Hth compared to that between Hox proteins and Gcm. In the case of Exd/Hth the Hox proteins collaborate in promoting chromatin accessibility, whereas in the case of Gcm, the chromatin accessibility state is predominantly controlled by Gcm.

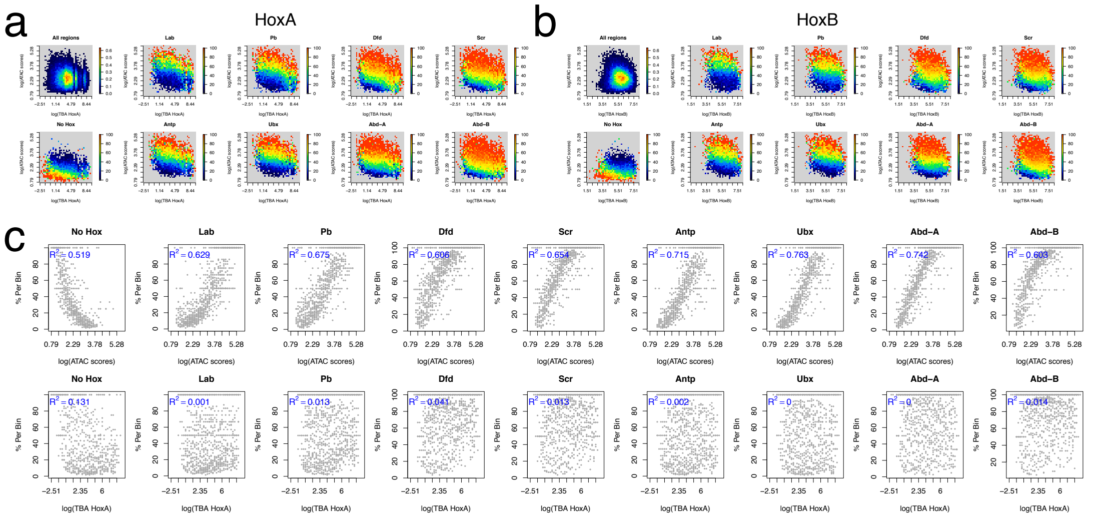

Figure S6 Hox occupancy is more strongly associated with binding region chromatin accessibility than with binding affinity.
Scatter plots of chromatin accessibility (log[ATAC scores]) versus binding affinity (log[TBA HoxA]) for chromatin regions classified as “open”. Open chromatin regions were divided into 200bp tiles and the mean ATAC score and TBA for HoxA PWM (a) or HoxB PWM (b) calculated per tile. The log of these scores was then linearly binned into 40 bins on each axis. For the “All regions” plot the heatmap shows the density distribution. For the other plots, the heatmap shows the percentage of tiles bound by the specified Hox protein per bin or for “No Hox” the percentage of tiles not bound by any Hox protein. (c) Scatter plots show the strong correlation of occupancy (% per bin) with chromatin accessibility (log[ATAC scores]; upper row) and the poor correlation with binding affinity ((log[TBA HoxA]; lower row). Data as in (a).

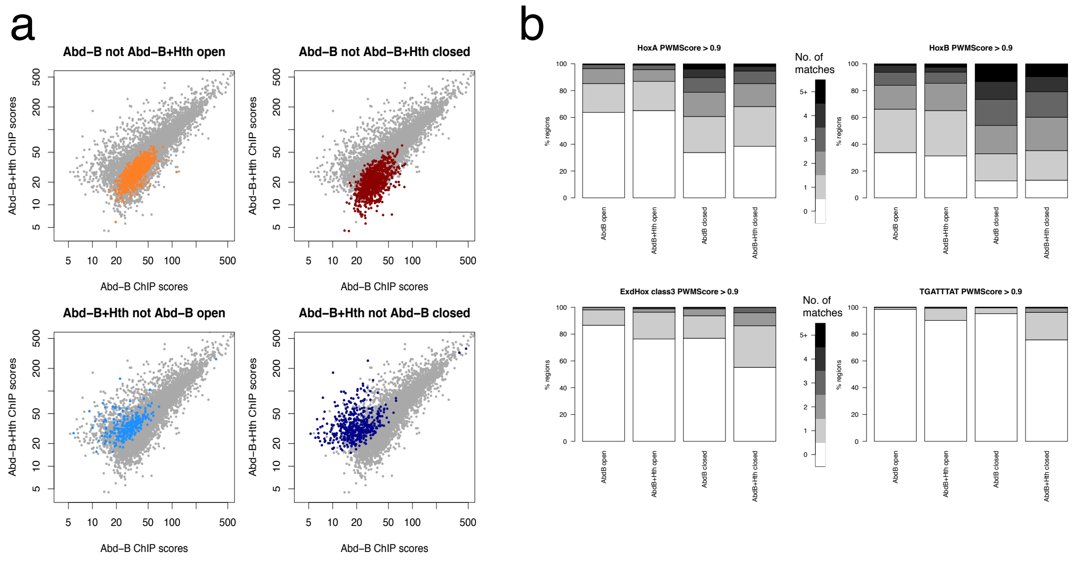

Figure S7 The presence of Exd/Hth leads to both enhanced and reduced Abd-B binding.
a: ChIP score scatterplots for Abd-B in presence (Abd-B + Hth) and absence (Abd-B) of Exd/Hth. The coloured populations. indicated above the plots, are

based on MACS peak calling (q1e-2) and the ChIP scores derived by creating a union peak set then calculating the mean ChIP score across the central 200bp. Orange: Abd-B peaks called only in the absence of Exd/Hth and in open chromatin; Brown: Abd-B peaks called only in the absence of Exd/Hth and in closed chromatin; Light Blue: Abd-B peaks called only in the presence of Exd/Hth and in open chromatin; Dark Blue: Abd-B peaks called only in the presence of Exd/Hth and in closed chromatin. b: Motif counting plots for the motifs named above the plots for the ChIP peak populations as in a). The Abd-B peaks called only in the absence of Exd/Hth and in closed chromatin (AbdB closed) show high number of matches for the HoxB motif whereas the Abd-B peaks called only in the presence of Exd/Hth and in closed chromatin (AbdB + Hth closed) show higher counts for the ExdHox class 3 PWM and Exd-Hox TGATTTAT motif.

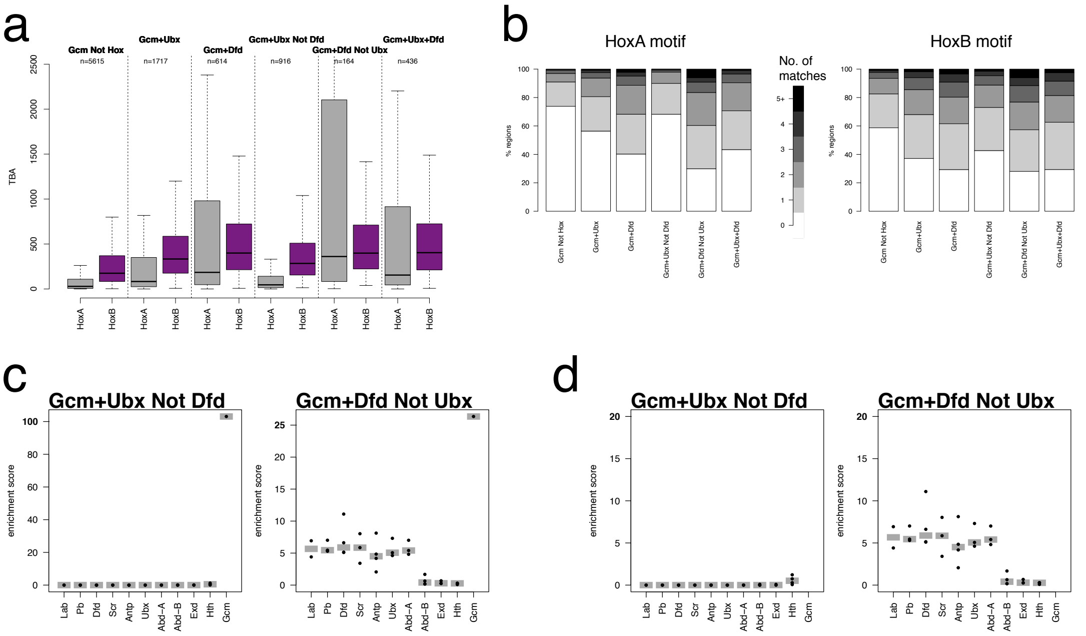

Figure S8 Comparison of Ubx and Dfd binding in presence of Gcm for regions in basal Kc167 closed chromatin.

a: Boxplot of TBA for the HoxA and HoxB motifs in 200bp regions which are in basal Kc167 closed chromatin corresponding to the populations (based on q1e-2 MACS peak calling) indicated above the plots. The population that binds Gcm and Dfd but not Ubx in the presence of Gcm (Gcm+Dfd Not Ubx) shows the highest TBA. b: Motif counting for HoxA and HoxB motifs on the populations in a) showing the highest counts for the population that binds Gcm and Dfd but not Ubx in the presence of Gcm (Gcm+Dfd Not Ubx). c: PWMEnrich analysis for the motif sets indicated on the x-axis for (left) the regions binding Gcm and Ubx but not Dfd in the presence of Gcm (Gcm+Ubx Not Dfd) and (right)) the regions binding Gcm and Dfd but not Ubx in the presence of Gcm (Gcm+Dfd Not Ubx). The plots are scaled to the Gcm motif enrichment. d: PWMEnrich plots as in c) but both plots scaled to enrichment score 20 to compare the Hox motif enrichments. The Gcm motif enrichment is off the scale.

Supplementary Tables

Table S1 ChIP-seq read overview

Transient Transfections

Sample	Total_reads	Mapped_reads	Platform
Lab_225	35,652,497	19,776,331	HiSeq 4000
Lab_226	31,656,390	17,331,670	HiSeq 4000
Lab_input	21,993,989	12,275,906	HiSeq 4000
Lab+Hth_228	19,535,480	10,340,548	HiSeq 4000
Lab+Hth_229	32,379,953	18,106,788	HiSeq 4000
Lab+Hth_input	19,029,087	10,947,693	HiSeq 4000
Pb_201	24,903,026	12,045,654	HiSeq 4000
Pb_202	24,425,098	11,872,562	HiSeq 4000
Pb_input	25,449,790	14,884,704	HiSeq 4000
Pb+Hth_203	32,955,638	13,326,705	HiSeq 4000
Pb+Hth_204	16,736,809	6,084,266	HiSeq 4000
Pb+Hth_input	32,830,552	19,085,144	HiSeq 4000
Dfd_113	20,422,023	11,993,655	HiSeq 4000
Dfd_114	18,352,596	11,186,142	HiSeq 4000
Dfd_input	43,905,501	27,275,300	HiSeq 4000
Dfd+Hth_117	35,673,391	22,902,065	HiSeq 4000
Dfd+Hth_118	31,982,490	20,279,888	HiSeq 4000
Dfd+Hth_input	54,438,852	33,037,109	HiSeq 4000
Scr_115	26,549,969	15,425,586	HiSeq 4000
Scr_116	25,966,588	14,805,387	HiSeq 4000
Scr_input	45,557,965	26,756,096	HiSeq 4000
Scr+Hth_119	20,753,220	12,635,134	HiSeq 4000
Scr+Hth_120	23,495,495	14,361,088	HiSeq 4000
Scr+Hth_input	31,207,186	18,740,677	HiSeq 4000
Antp_67	33,839,220	15,204,772	HiSeq 4000
Antp_68	42,079,289	18,791,811	HiSeq 4000
Antp_input	39,031,841	22,009,066	HiSeq 4000
Antp+Hth_75	28,360,324	12,993,786	HiSeq 4000
Antp+Hth_76	29,674,613	13,895,182	HiSeq 4000
Antp+Hth_input	21,860,819	13,107,646	HiSeq 4000
Ubx_1	26,255,388	12,094,751	HiSeq 2500
Ubx_2	27,539,748	13,518,594	HiSeq 2500
Ubx_input	48,617,397	27,131,432	HiSeq 2500
Ubx+Hth_1	25,473,053	11,488,879	HiSeq 2500
Ubx+Hth_2	36,697,607	16,089,206	HiSeq 2500
Ubx+Hth_input	25,155,474	14,512,496	HiSeq 2500
Abd-A_49	18,734,401	10,471,829	HiSeq 2500
Abd-A_50	23,927,894	12,894,433	HiSeq 2500
Abd-A_input	21,996,208	12,532,916	HiSeq 2500

Abd-A+Hth_53	23,305,535	12,221,375	HiSeq 2500
Abd-A+Hth_54	16,228,122	8,881,775	HiSeq 2500
Abd-A_input	34,576,407	20,530,123	HiSeq 2500
Abd-B_51	22,788,335	12,470,631	HiSeq 2500
Abd-B_52	23,583,829	13,700,974	HiSeq 2500
Abd-B_input	21,996,208	12,532,916	HiSeq 2500
Abd-B+Hth_55	22,523,431	13,915,019	HiSeq 2500
Abd-B+Hth_56	22,403,332	7,508,223	HiSeq 2500
Abd-B_input	34,576,407	20,530,123	HiSeq 2500
Hth_209	38,180,841	21,305,298	HiSeq 4000
Hth_210	37,150,247	20,663,723	HiSeq 4000
Hth_input	29,208,741	16,401,950	HiSeq 4000
Exd+Hth_232	28,385,162	17,462,671	HiSeq 4000
Exd+Hth_233	23,369,536	14,523,255	HiSeq 4000
Exd+Hth_input	32,952,245	18,571,046	HiSeq 4000

Stable Cell Lines

**Sample**	**Total_reads**	**Mapped_reads**	**Platform**
Dfd_247	32,843,529	18,779,761	HiSeq 4000
Dfd_248	17,095,829	10,292,158	HiSeq 4000
Dfd_input	27,181,802	16,427,314	HiSeq 4000
Dfd+Hth_243	21,869,632	14,114,182	HiSeq 4000
Dfd+Hth_244	25,725,965	16,369,234	HiSeq 4000
Dfd+Hth_input	23,240,584	14,492,712	HiSeq 4000
Dfd+Gcm_249	36,208,630	21,210,268	HiSeq 4000
Dfd+Gcm_250	20,584,442	12,196,295	HiSeq 4000
Dfd+Gcm_input	41,719,566	25,980,366	HiSeq 4000
Ubx_217	40,574,216	22,651,116	HiSeq 4000
Ubx_218	33,836,429	19,965,122	HiSeq 4000
Ubx_input	23,514,868	14,304,826	HiSeq 4000
Ubx+Hth_213	37,082,776	21,381,969	HiSeq 4000
Ubx+Hth_214	32,117,571	18,761,644	HiSeq 4000
Ubx+Hth_input	26,843,880	16,594,979	HiSeq 4000
Ubx+Gcm_235	45,711,964	26,450,967	HiSeq 4000
Ubx+Gcm_236	44,458,658	26,238,573	HiSeq 4000
Ubx+Gcm_input	41,935,371	24,920,987	HiSeq 4000
Abd-B_239	39,832,402	26,838,262	HiSeq 4000
Abd-B_240	72,131,533	45,950,537	HiSeq 4000
Abd-B_input	37,184,024	23,188,692	HiSeq 4000
Abd-B+Hth_241	33,136,444	22,093,185	HiSeq 4000
Abd-B+Hth_242	22,183,688	14,712,517	HiSeq 4000
Abd-B+Hth_input	34,244,185	21,439,677	HiSeq 4000
Gcm_219	38,041,560	23,403,544	HiSeq 4000
Gcm_220	38,754,938	23,227,820	HiSeq 4000

Gcm_input	39,285,389	23,608,832	HiSeq 4000
Hth_215	42,237,064	26,788,395	HiSeq 4000
Hth_216	36,857,352	23,428,980	HiSeq 4000
Hth_input	24,560,848	15,060,156	HiSeq 4000

Table S2 ChIP-Seq binding region numbers

Union gives the number of binding regions present in both replicates.

Transient transfections

	q1e-2			q1e-10		
**Sample**	**rep1**	**rep2**	**union**	**rep1**	**rep2**	**union**
Lab	4206	4742	**3835**	1790	2089	**1704**
Lab+Hth	5406	5922	**4827**	2493	2741	**2325**
Pb	7204	6519	**5598**	3301	2903	**2682**
Pb+Hth	7605	6204	**5708**	3887	2990	**2883**
Dfd	9773	9365	**8467**	5703	5102	**4782**
Dfd+Hth	13195	12952	**12054**	9731	9510	**8958**
Scr	8488	8576	**7355**	4700	4793	**4127**
Scr+Hth	7616	8581	**6603**	4257	5285	**3797**
Antp	3970	5468	**3523**	921	1451	**846**
Antp+Hth	3289	4728	**2887**	1154	1704	**1043**
Ubx	5271	4372	**3752**	1581	1209	**1083**
Ubx+Hth	6473	7619	**5684**	2428	3112	**2242**
Abd-A	6251	7242	**5487**	2412	3203	**2234**
Abd-A+Hth	7525	7867	**6528**	3416	3617	**3040**
Abd-B	11414	10906	**9786**	6915	6133	**5685**
Abd-B+Hth	10976	10144	**9044**	6444	5476	**5027**
Hth	6572	5872	**5373**	3551	2921	**2781**
Exd+Hth	11451	11590	**10409**	8623	8383	**7695**

Stable Cell Lines

	q1e-2			q1e-10		
**Sample**	**rep1**	**rep2**	**union**	**rep1**	**rep2**	**union**
Exd+Hth_stable	11451	11590	**10409**	8623	8383	**7695**
Gcm_stable	12301	11863	**10765**	6683	6125	**5831**
Hth_stable	9891	10045	**8999**	6713	7238	**6225**
Dfd_stable	12332	11500	**10380**	6261	5566	**5145**
Dfd+Hth_stable	11156	11699	**9867**	6464	7260	**5981**
Dfd+Gcm_stable	10261	10370	**8893**	4533	4506	**3924**
Ubx_stable	6251	6019	**5281**	2206	2066	**1833**
Ubx+Hth_stable	5406	6852	**4885**	2415	3422	**2269**
Ubx+Gcm_stable	10035	11039	**8846**	4612	6140	**4275**
Abd-B_stable	14696	14839	**13511**	11371	11222	**10375**
Abd-B+Hth_stable	15441	14902	**13741**	11152	10050	**9418**

Table S3 Stable cell lines ATAC-seq read overview

ATAC_Kc represent standard Kc167 cells, all other samples are stable Kc-cell lines containing pMT-puro-Hox plasmids.

i = induced (CuSO4), n = non-induced

Sample	Total_reads	Mapped_reads	Platform
ATAC_Kc_7	112,702,038	90,189,400	HiSeq 4000
ATAC_Kc_10	113,325,609	87,818,612	HiSeq 4000
ATAC_Kc_11	131,356,618	102,734,912	HiSeq 4000
ATAC_Dfd_i_114	33,979,005	26,174,422	HiSeq 4000
ATAC_Dfd_i_115	47,498,366	35,298,598	HiSeq 4000
ATAC_Dfd_n_116	8,106,401	5,926,680	HiSeq 4000
ATAC_Dfd_n_117	16,827,993	12,395,692	HiSeq 4000
ATAC_Dfd+Hth_i_106	20,645,066	15,412,147	HiSeq 4000
ATAC_Dfd+Hth_i_107	33,393,909	25,018,382	HiSeq 4000
ATAC_Dfd+Hth_n_108	45,517,692	33,527,834	HiSeq 4000
ATAC_Dfd+Hth_n_109	36,571,843	27,512,683	HiSeq 4000
ATAC_Dfd+Gcm_i_118	19,123,450	14,542,498	HiSeq 4000
ATAC_Dfd+Gcm_i_119	31,521,343	23,755,454	HiSeq 4000
ATAC_Dfd+Gcm_n_120	32,482,773	23,479,769	HiSeq 4000
ATAC_Dfd+Gcm_n_121	22,607,002	16,957,603	HiSeq 4000
ATAC_Ubx_i_62	33,125,682	23,727,431	HiSeq 4000
ATAC_Ubx_i_63	47,570,852	34,116,016	HiSeq 4000
ATAC_Ubx_n_64	13,589,247	10,291,131	HiSeq 4000
ATAC_Ubx_n_65	24,444,782	18,040,490	HiSeq 4000
ATAC_Ubx+Hth_i_54	34,488,251	24,207,832	HiSeq 4000
ATAC_Ubx+Hth_i_55	30,566,866	23,181,127	HiSeq 4000
ATAC_Ubx+Hth_n_56	50,532,615	38,846,046	HiSeq 4000
ATAC_Ubx+Hth_n_57	33,874,296	25,808,470	HiSeq 4000
ATAC_Ubx+Gcm_i_90	47,304,001	37,298,997	HiSeq 4000
ATAC_Ubx+Gcm_i_91	52,695,324	43,620,118	HiSeq 4000
ATAC_Ubx+Gcm_n_92	30,341,723	22,678,298	HiSeq 4000
ATAC_Ubx+Gcm_n_93	13,620,559	10,014,547	HiSeq 4000
ATAC_Abd-B_i_98	22,418,822	17,167,407	HiSeq 4000
ATAC_Abd-B_i_99	24,413,883	19,340,938	HiSeq 4000
ATAC_Abd-B_n_100	20,580,779	15,982,847	HiSeq 4000
ATAC_Abd-B_n_101	27,866,139	21,673,485	HiSeq 4000
ATAC_Abd-B+Hth_i_102	41,645,070	34,657,116	HiSeq 4000
ATAC_Abd-B+Hth_i_103	23,886,047	18,624,131	HiSeq 4000
ATAC_Abd-B+Hth_n_104	19,484,964	14,958,616	HiSeq 4000
ATAC_Abd-B+Hth_n_105	26,325,518	19,788,572	HiSeq 4000
ATAC_Gcm_i_86	30,462,152	24,328,017	HiSeq 4000
ATAC_Gcm_i_87	18,888,376	15,277,774	HiSeq 4000

ATAC_Gcm_n_88	26,542,697	20,168,748	HiSeq 4000
ATAC_Gcm_n_89	12,643,646	9,371,579	HiSeq 4000
ATAC_Hth_i_58	31,491,896	22,619,681	HiSeq 4000
ATAC_Hth_i_59	27,549,490	20,662,463	HiSeq 4000
ATAC_Hth_n_60	31,034,936	23,887,936	HiSeq 4000
ATAC_Hth_n_61	34,868,300	27,248,785	HiSeq 4000

Table S4 Cofactor-enhanced binding analysis of transient data in Hox group peak regions

EdgeR results of ChIP-seq data locating enhanced bound regions in the presence of Hth using fdr <= 0.01 and logFC > 1. "Common bound" are peak regions bound at similar levels (or do not have a significant fdr). "Hox enhanced bound" (these may represent the noise in the genomic data) regions are fdr <= 0.01, but have a logFC < -1.

	Hox+Hth enhanced bound	Common bound	Hox enhanced bound
**Lab+Hth vs Lab**	**1248**	2487	225
**Pb+Hth vs Pb**	**463**	5600	58
**Dfd+Hth vs Dfd**	**1113**	8264	239
**Scr+Hth vs Scr**	**391**	7769	94
**Antp+Hth vs Antp**	**1140**	1241	208
**Ubx+Hth vs Ubx**	**428**	3077	14
**Abd-A+Hth vs Abd-A**	**282**	5935	8
**Abd-B+Hth vs Abd-B**	**351**	9688	87

Table S5 Increased chromatin accessibility analysis

EdgeR results of ATAC-seq data locating increased accessible regions in the 21,002 Hox group peaks - stable regions comparing induced (CuSO4) versus non-induced samples. Increased accessiblity regions have fdr <= 0.01 and logFC > 1.5. Decreased accessibility regions which have fdr <= 0.01 and logFC < -1.5 and may represent noise in the genomic data.

	increased accessibility	decreased accessibility
Dfd induced vs Dfd non-induced	430	122
Ubx induced vs Ubx non-induced	47	128
Abd-B induced vs Abd-B non-induced	832	103
Hth induced vs Hth non-induced	17	14
